# D‐Mannose Alleviates Type 2 Diabetes and Rescues Multi‐Organ Deteriorations by Controlling Release of Pathological Extracellular Vesicles

**DOI:** 10.1002/EXP.20240133

**Published:** 2025-08-25

**Authors:** Sha Zhang, Kai Zhang, Chen‐Xi Zheng, Ying‐Feng Gao, Guo‐Rong Deng, Xu Zhang, Yuan Yuan, Ting Jia, Si‐Yuan Tang, Guang‐Xiang He, Zhen Gong, Na Zhao, Bo Ma, Hua Tian, Hong Zhang, Zhe Li, Yong‐Chang Di‐Wu, Yi‐Han Liu, Liang Kong, Jing Ma, Yan Jin, Bing‐Dong Sui

**Affiliations:** ^1^ Department of Traditional Chinese Medicine The First Affiliated Hospital of Fourth Military Medical University Xi'an China; ^2^ State Key Laboratory of Oral & Maxillofacial Reconstruction and Regeneration National Clinical Research Center for Oral Diseases Shaanxi International Joint Research Center for Oral Diseases Center for Tissue Engineering School of Stomatology The Fourth Military Medical University Xi'an China; ^3^ College of Basic Medicine Shaanxi University of Chinese Medicine Xianyang China; ^4^ Department of Oral and Maxillofacial Surgery School of Stomatology The Fourth Military Medical University Xi'an China; ^5^ Xi'an Key Laboratory of Stem Cell and Regenerative Medicine Institute of Medical Research Northwestern Polytechnical University Xi'an China; ^6^ Department of Critical Care Medicine the Second Affiliated Hospital of Xi'an Jiaotong University Xi'an China; ^7^ The First Clinical Medical College Shaanxi University of Chinese Medicine Xianyang China; ^8^ Military Medical Innovation Center The Fourth Military Medical University Xi'an China; ^9^ Academy of Military Medical Sciences Beijing China; ^10^ Department of Diabetes School of Translational Medicine Monash University Melbourne Australia; ^11^ Medical Experiment Center Shaanxi University of Chinese Medicine Xianyang China; ^12^ Department of Stomatology the First Medical Center Chinese PLA General Hospital Beijing China

**Keywords:** D‐mannose, type 2 diabetes, fatty liver, macrophage, extracellular vesicles, osteoporosis

## Abstract

Type 2 diabetes (T2D) is a prevalent metabolic disease inducing alterations of multiple organ systems with currently no cure. Extracellular vesicles (EVs) have been increasingly noticed as one critical paracrine communicator inducing insulin resistance and metabolic disorders in T2D, but clinically available pharmaceuticals for controlling pathological EV release is lacking. Here, we discover that the natural monosaccharide D‐mannose exists with an altered level in the db/db mouse T2D model. Intriguingly, oral administration of D‐mannose with the drinking water safely ameliorates diabetic symptoms in db/db mice. D‐mannose administration does not critically regulate the gut microbiome and circulatory T lymphocytes in treating T2D, while administrated D‐mannose rapidly accumulates in the liver, alleviates hepatic steatosis and rescues insulin resistance. Regarding the mechanism, the T2D pathological EVs released by macrophages are targeted and reduced by D‐mannose, which metabolically inhibits CD36 expression and restores function of hepatocytes. Importantly, by regulating macrophage EV release, D‐mannose administration reveals extra‐hepatic benefits and retards diabetic bone loss. Taken together, our findings unveil D‐mannose as a candidate T2D therapeutic and highlight sugars governing intercellular EV crosstalk, paving an avenue for pharmaceutical T2D approaches with amelioration of multi‐organ deteriorations.

## Graphical abstract

In this study, Zhang and colleagues show that drinking‐water supplementation of D‐mannose serves as an effective and potential therapeutic of type 2 diabetes, with improvement of both liver health and bone mass. The effect is exerted through suppressing macrophage release of extracellular vesicles based on metabolic control of CD36 expression. These findings shed light on translational pharmaceutical strategies of type 2 diabetes.

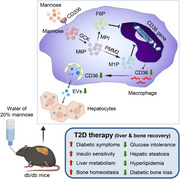

## 1 Introduction

Type 2 diabetes (T2D) is a chronic progressive metabolic disease with high and increasing global prevalence, which represents a major cause of morbidity and even mortality [1, 2]. Particularly, epidemiological studies have demonstrated that individuals diagnosed with T2D are at increased risks of developing non‐alcoholic fatty liver disease (NAFLD) and osteoporotic fractures, significantly among its life‐threatening multi‐organ deteriorations and complications [3, 4, 5]. Of note, T2D is characterized by or associated with a wide spectrum of pathologies, including obesity, insulin resistance, and macrophage‐mediated chronic low‐grade inflammation [1, 6]. Particularly, pro‐inflammatory activation of tissue macrophages in the obese condition is known to release multiple cytokines, such as tumor necrosis factor‐alpha (TNF‐α), interleukin‐1beta (IL‐1β) and galectin‐3, as well as extracellular vesicles (EVs), membranous nanoparticles for intercellular communication [7, 8], to impair insulin sensitivity and induce metabolic alterations, including hepatic steatosis and bone loss, in target organs [9, 10, 11, 12]. However, current anti‐inflammatory therapies have limited, conditional or suboptimal effects on glycemic control and ameliorating insulin resistance in T2D patients [13, 14, 15]. Furthermore, although the role of endogenous EVs in human health and disease is emergingly being revealed, clinically available pharmaceuticals for controlling pathological EV release is still lacking [16, 17, 18, 19, 20]. Therefore, there remains an unmet need to unravel feasible pharmacological targets of restraining macrophage‐based paracrine crosstalk in T2D and develop therapeutic approaches accordingly.

Integrated analyses by combining cell‐specific metabolic, transcriptional regulatory and protein‐protein interaction networks in human have identified increased levels of plasma mannose in obese subjects and discovered significant correlations between high circulating mannose concentrations with insulin resistance and the incidence of T2D in large prospective cohorts [21, 22]. Notably, it is unclear whether this change is a concomitant or causative cause of T2D. D‐mannose, a natural C‐2 epimer of glucose, is a monosaccharide found in plants and fruits and exists in human blood at a concentration less than one‐fiftieth of glucose, which contributes to protein glycosylation and represents an alternative energy source [23, 24, 25]. Importantly, D‐mannose administration orally via drinking water at supraphysiological levels has been proved effective to treat congenital and infectious human diseases [26, 27], which has also been reported useful to treat T lymphocyte‐ and macrophage‐associated immunopathologies and improve glucose and lipid metabolism in mice [28, 29, 30, 31]. Therefore, D‐mannose might exert beneficial effects on T2D despite the increased plasma level, but whether and how D‐mannose regulates T2D development remains elusive. Notably, the release of EVs has been emergingly revealed to be regulated by sugars and glycosylation [32]. Further investigations on the potential effects of D‐mannose modulating EV release under pathophysiological condition may provide additional interesting mechanisms supporting its translational promise.

In this study, we aim to investigate that whether and how D‐mannose may regulate T2D with potential benefits on multiple diseased organs. We adopted the genetically obese, leptin receptor‐deficient db/db mice as a T2D model [33]. Indeed, leptin receptor signaling has been revealed as a critical regulator of macrophage inflammation in obesity and insulin resistance, which prevents overloaded metabolic stress of macrophages in a lipid‐rich microenvironment, but evidence of leptin receptor signaling regulating macrophage EVs is lacking [34, 35, 36]. Through a series of experiments, we were able to decipher the effect and mechanism of D‐mannose regulating T2D.

## 2 Results

### 2.1 Altered Mannose Metabolism is Associated With T2D in Genetically Obese db/db Mice

**FIGURE 1:**
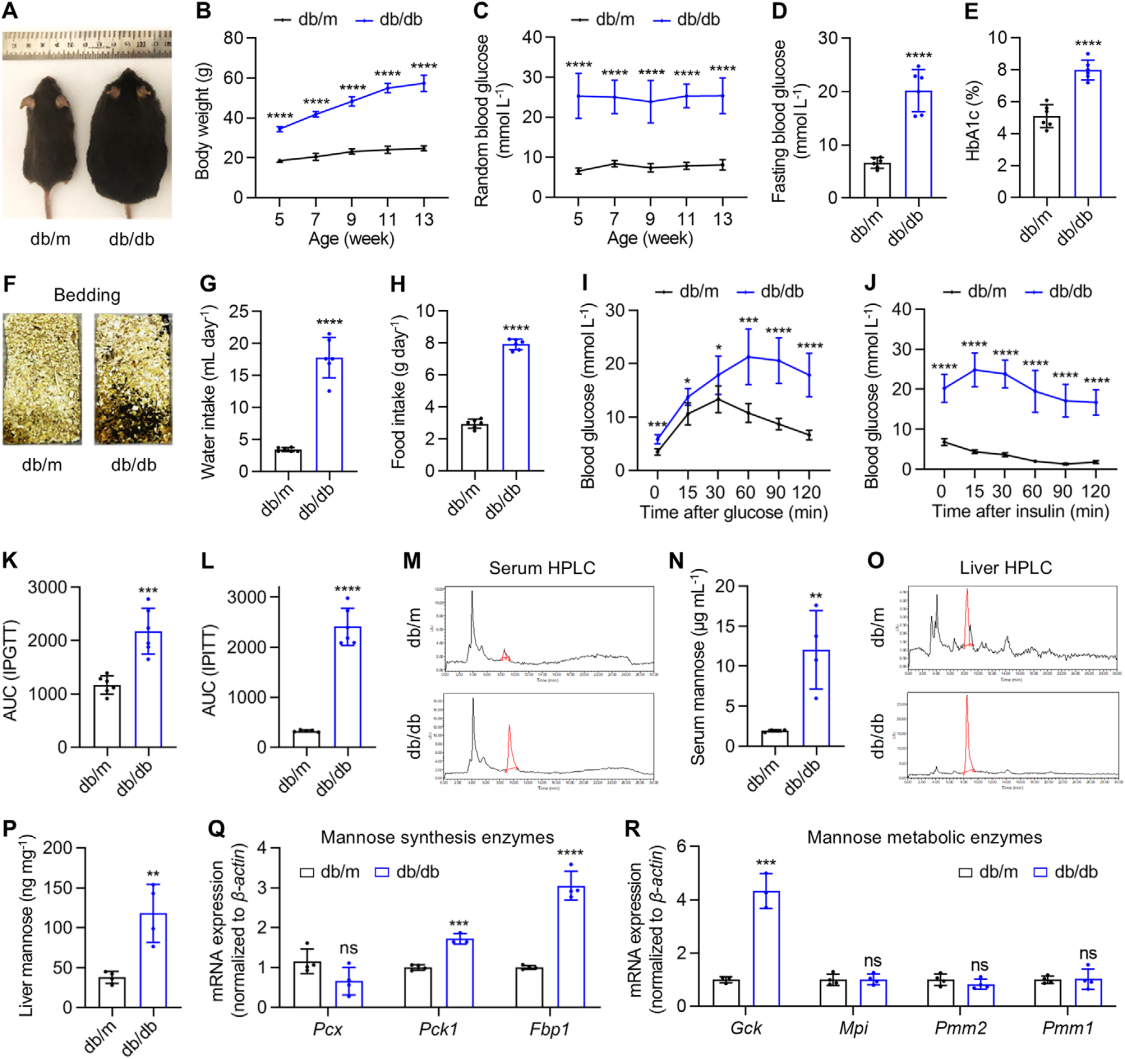
Altered mannose metabolism is associated with type 2 diabetes (T2D) in genetically obese db/db mice. (A) The gross view image of db/m and db/db male mice at 13‐week old. (B) Body weight changes of db/m and db/db male mice, *n* = 6. (C) Random blood glucose levels sampled from the tail vein, *n* = 6. (D) Blood glucose levels after fasting for 6 h, *n* = 6. (E) Blood hemoglobin A1c (HbA1c) levels, *n* = 6. (F) Cage bedding after 5 days of 4 mice in each cage. (G) Average water intake of a single mouse per day, *n* = 6. (H) Average food intake of a single mouse per day, *n* = 6. (I) The intraperitoneal glucose tolerance test (IPGTT) after 20‐h fasting and 1 g kg^−1^ glucose injection. *n* = 5–6. (J) The intraperitoneal insulin tolerance test (IPITT) after 6‐h fasting and 2 IU kg^−1^ insulin injection, *n*=5–6. (K) Area under curve (AUC) analysis of IPGTT, *n*=5–6. (L) AUC analysis of IPITT, *n* = 5–6. (M) High performance liquid chromatography (HPLC) analysis of serum samples with red peaks indicating mannose. (N) Serum mannose levels quantified by HPLC, *n* = 4. (O) HPLC analysis of liver contents with red peaks indicating mannose. (P) Liver mannose levels quantified by HPLC, *n* = 4. (Q) Quantitative real‐time polymerase chain reaction (qRT‐PCR) analysis of mannose synthesis enzyme expression in the liver, *n* = 3–4. (R) qRT‐PCR analysis of mannose metabolic enzyme expression in the liver, *n* = 3–4. Mean ± SD. *, *p* < 0.05; **, *p* < 0.01; ***, *p* < 0.001; ****, *p* < 0.0001; ns, *p* > 0.05. Two‐tailed unpaired Student's *t* test.

To begin, phenotypic examination of db/db mice with their age‐ and sex‐matched db/m control was performed (Figure S1). Expectedly, db/db mice developed increasingly higher body weight from 5 to 13 weeks of age (i.e., the experimental period) compared to db/m mice (Figure 1A,B). Furthermore, during the experimental period, db/db mice exhibited high random blood glucose levels over the diabetic criteria of 16.8 mmol L^−1^ (about 300 mg dL^−1^), with also high fasting blood glucose levels over the diabetic criteria of 11.1 mmol L^−1^ (about 200 mg dL^−1^) (Figure 1C,D). Moreover, percentages of glycated hemoglobin A1c (HbA1c) in blood, which indicates long‐term glucose status, were detected over the diabetic criteria of 6.5% in db/db mice (Figure 1E). Notably, db/db mice resembled clinical diabetic symptoms of polyuria, polydipsia and polyphagia, showing wetter bedding and higher amount of water and food intake than db/m mice (Figure 1F–H). Importantly, db/db mice demonstrated reduced tolerance to intraperitoneal glucose injection and decreased insulin sensitivity, as evidenced by intraperitoneal glucose and insulin tolerance test (IPGTT and IPITT) experiments (Figure 1I–L).

For mannose levels, we applied high performance liquid chromatography (HPLC) to examine serum samples (Figure 1M). Results revealed that the average levels of mannose in serum of db/db mice were 6‐fold higher than db/m mice, reaching 12 µg mL^−1^ (beyond 60 µm) with statistical significance (Figure 1N). HPLC examination of mannose contents in the liver, the major organ of physiological mannose synthesis and metabolic consumption [21, 37], also demonstrated over 2‐fold increase in db/db mice compared to db/m mice (Figure 1O,P). Accordingly, quantitative real‐time polymerase chain reaction (qRT‐PCR) analysis of mRNA expression of mannose synthesis enzymes in the liver, namely the critical genes for gluconeogenesis [38], *Pyruvate carboxylase* (*Pcx*), *Phosphoenolpyruvate carboxykinase 1* (*Pck1*) and *Fructose bisphosphatase 1* (*Fbp1*), revealed upregulation of *Pck1* and *Fbp1* in db/db mice (Figure 1Q). Interestingly, mRNA expression levels of mannose metabolic enzymes in the liver [25], namely *Glucokinase* (*Gck*), *Mannose phosphate isomerase (Mpi)*, *Phosphomannomutase 2 (Pmm2)* and *Pmm1*, showed upregulated or paralleled levels in db/db mice (Figure 1R). These results indicated maintained capability of metabolizing mannose in T2D, despite the non‐specific acceleration of mannose production attributed to promoted gluconeogenesis. Taken together, these findings suggest that altered mannose metabolism is associated with T2D in db/db mice.

### 2.2 Oral Administration of D‐Mannose With the Drinking Water Safely Ameliorates T2D in db/db Mice

**FIGURE 2:**
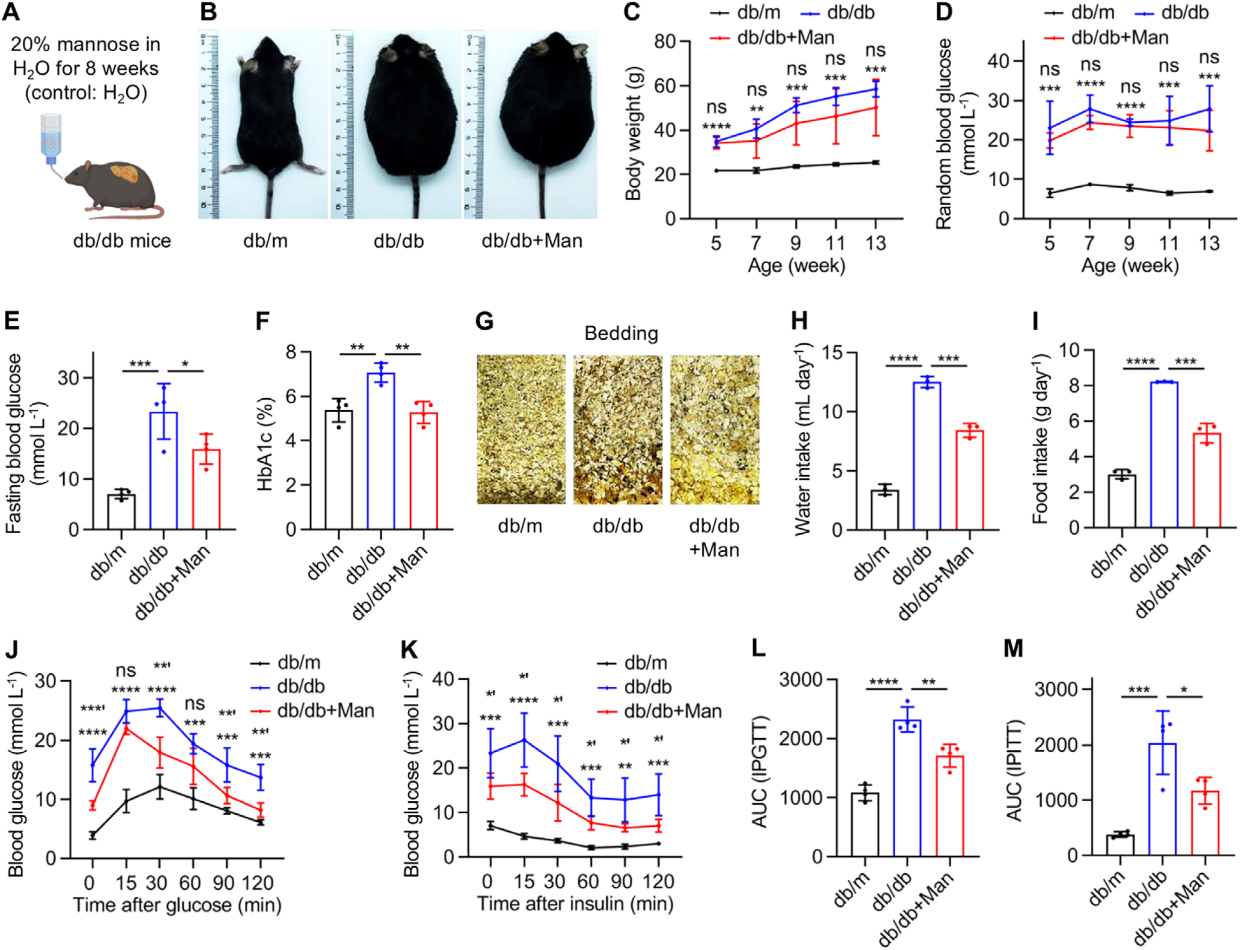
Drinking‐water supplementation of D‐mannose safely ameliorates type 2 diabetes (T2D) in db/db mice. (A) The diagram showing drinking‐water supplementation of D‐mannose (Man). (B) Gross view images of mice at 13‐week old. (C) Body weight changes of mice, *n* = 4–5. **, *** or ****, db/db compared to db/m. ns, db/db+Man compared to db/db. (D) Random blood glucose levels sampled from the tail vein, *n* = 4. *** or ****, db/db compared to db/m. ns, db/db+Man compared to db/db. (E) Blood glucose levels after fasting for 6 h, *n* = 4. (F) Blood hemoglobin A1c (HbA1c) levels, *n* = 4. (G) Cage bedding after 5 days of 4 mice in each cage. (H) Average water intake of a single mouse per day, *n* = 3. (I) Average food intake of a single mouse per day, *n* = 3. (J) The intraperitoneal glucose tolerance test (IPGTT) after 20‐h fasting and 1 g kg^−1^ glucose injection, *n* = 4. *** or ****, db/db compared to db/m. **′, ***′ or ns, db/db+Man compared to db/db. (K) The intraperitoneal insulin tolerance test (IPITT) after 6‐h fasting and 2 IU kg^−1^ insulin injection, *n* = 4. **, *** or ****, db/db compared to db/m. *′, db/db+Man compared to db/db. (L) Area under curve (AUC) analysis of IPGTT, *n* = 4. (M) AUC analysis of IPITT, *n* = 4. Mean ± SD. * and *’, *p* < 0.05; ** and **′, *p* < 0.01; *** and ***′, *p* < 0.001; ****, *p* < 0.0001; ns, *p* > 0.05. One‐way ANOVA with Turkey's post‐hoc test.

The above findings indicate elevated mannose levels as a concomitant phenomenon in the course of T2D. Also, the maintained capability of metabolizing mannose in T2D and the previously documented multiple beneficial effects of D‐mannose enlightened us to investigate whether D‐mannose administration would help alleviate T2D in db/db mice. We adopted the method of supraphysiological mannose supplementation in drinking‐water (20% or 0.2 g mL^−1^, equal to 1.1 mol L^−1^) [28] to treat db/db mice during the 8‐week experimental period (Figure 2A). Intriguingly, we found that drinking‐water supplementation of D‐mannose did not significantly affect the body weight gain (Figure 2B,C) or the random blood glucose levels (Figure 2D) of db/db mice, but it indeed reduced the fasting blood glucose levels and rescued the blood HbA1c percentages of db/db mice (Figure 2E,F), indicating efficacy related to stimulation and in the long‐term. Furthermore, oral administration of D‐mannose ameliorated the diabetic symptoms in db/db mice, showing controlled urine output and suppressed water and food intake (Figure 2G–I). Importantly, D‐mannose administration improved the glucose tolerance condition and promoted insulin sensitivity of db/db mice, as evidenced by IPGTT and IPITT, suggesting the therapeutic effects (Figure 2J–M). In addition, histological analysis across multiple organs identified limited influence of D‐mannose on the heart, lung, kidney and spleen morphology (Figure S2A–H), despite reduced organ weights in db/db mice, with also limited effects on the fat mass, lean mass or body fluid composition of db/db mice (Figure S1I–L). Taken together, these findings indicate that oral administration of D‐mannose with the drinking water safely ameliorates T2D in db/db mice.

### 2.3 D‐mannose Administration Does Not Critically Regulate the Gut Microbiome and Circulatory T Lymphocytes in Treating T2D

**FIGURE 3:**
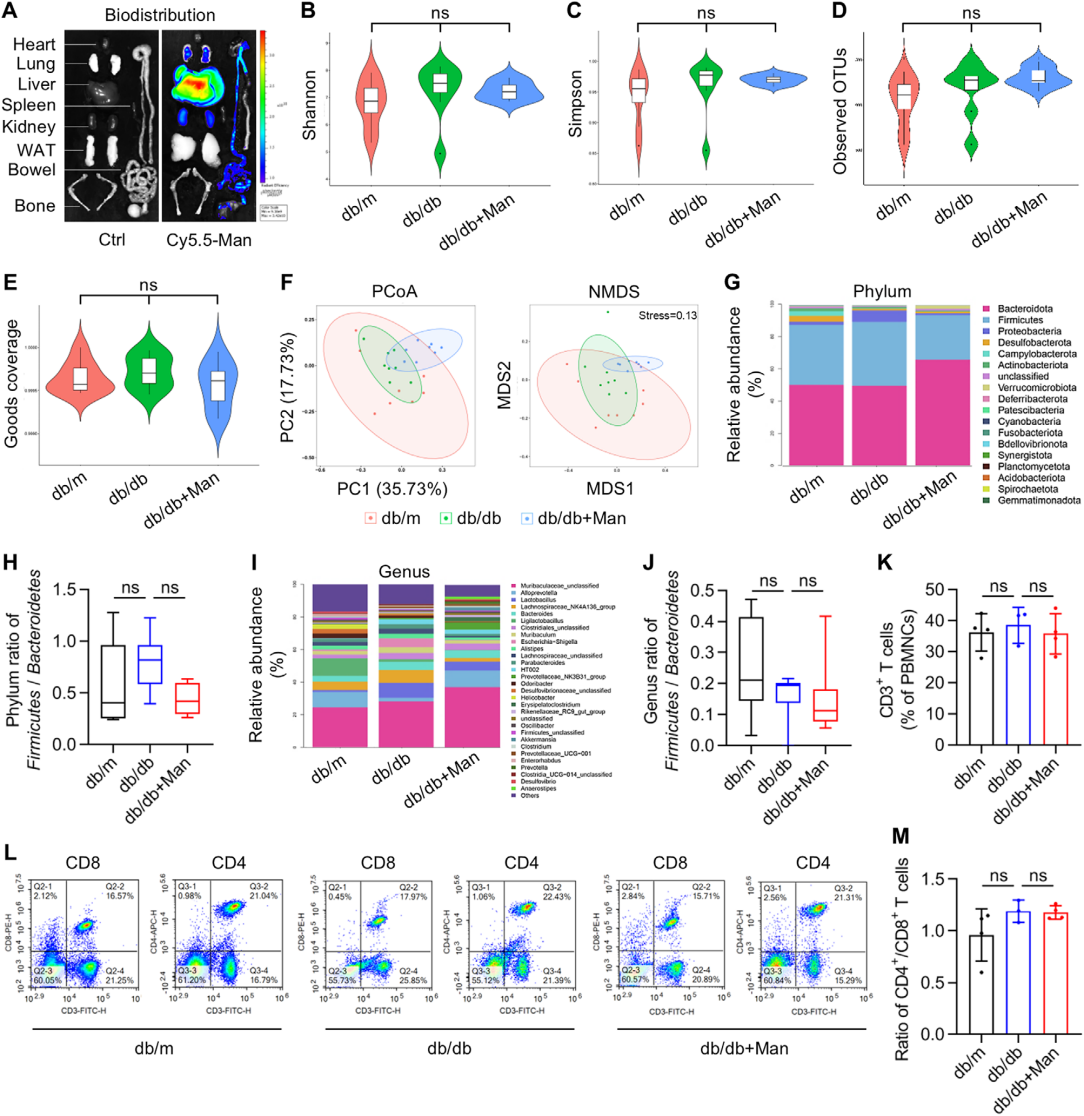
D‐mannose administration exerts limited effects on the gut microbiome and peripheral blood T lymphocytes. (A) Biodistribution of Cy5.5‐labeled fluorescent mannose (Man) after oral administration for 24 h. WAT, white adipose tissue. (B) The violin plot showing the Shannon index of α diversity in 16S rRNA sequencing of the gut microbiome, *n* = 8. (C) The violin plot showing the Simpson index of α diversity, *n* = 8. (D) The violin plot showing the observed operational taxonomic units (OTUs) index of α diversity, *n* = 8. (E) The violin plot showing the goods coverage index of α diversity, *n* = 8. (F) Principal coordinates analysis (PCoA) and nonmetric multidimensional scaling (NMDS) analyses of β diversity, *n* = 8. (G) The stacked bar chart showing the relative abundance of phyla. (H) Quantification of ratio of *Firmiccutes* over *Bacteroidetes* at the phylum level, *n* = 6–8. (I) The stacked bar chart showing the relative abundance of genera. (J) Quantification of ratio of *Firmiccutes* over *Bacteroidetes* at the genus level, *n* = 6–8. (K) CD3^+^ T cell percentages in peripheral blood mononucleated cells (PBMNCs) analyzed by flow cytometry, *n* = 3–4. (L) Flow cytometric analysis of CD8^+^ and CD4^+^ percentages in peripheral blood CD3^+^ T cells. (M) Quantification of ratio of CD4^+^ T cells over CD8^+^ T cells in the peripheral blood, *n* = 3–4. ns, *p* > 0.05. Box (25th, 50th, and 75th percentiles) and whisker (range) and Kruskal–Wallis test (B–E, H, and J), or mean ± SD and one‐way ANOVA with Turkey's post‐hoc test (K and M).

Next, we investigated how D‐mannose may ameliorate T2D in db/db mice. Previous studies have reported that mannose administration in water regulates gut microbiome and prevents high‐fat diet (HFD)‐induced obesity, and that drinking‐water supplementation of D‐mannose suppresses T lymphocytes‐based immunopathology for autoimmune type 1 diabetes (T1D) therapy [28, 30]. With this knowledge, we first performed biodistribution analysis of Cy5.5‐labeled fluorescent mannose after oral administration in db/db mice. Data showed that exogenous mannose rapidly accumulated in the liver of db/db mice during a 24‐h period, suggesting successful entrance into circulation (Figure 3A and Figure S3A). Notably, the bowel and the kidney were also fluorescently labeled, which corresponded to absorption and excretion of mannose respectively through the intestine and via the urine (Figure 3A). Accordingly, we examined that whether the gut microbiome in db/db mice was affected by mannose administration. 16S rRNA sequencing (Table S1) and related indexes of α diversity showed limited influence of D‐mannose on the gut microbiome of db/db mice (Figure 3B–E), which were further supported by the principal coordinates analysis (PCoA) and the nonmetric multidimensional scaling (NMDS) analyses of β diversity (Figure 3F). Moreover, the relative abundance of microbiome compositions with quantifications at phylum and genus levels of *Firmicutes* over *Bacteroidetes* ratio, a relevant index of gut dysbiosis in obese individuals [39], revealed no significant effects of D‐mannose administration (Figure 3G–J). Considering that D‐mannose entered into the blood after intestinal absorption, we then examined that whether peripheral blood T cells were affected. Flow cytometric analysis demonstrated that neither the total CD3^+^ T cell percentages among the peripheral blood mononucleated cells (PBMNCs) (Figure 3K) nor the CD4^+^/CD8^+^ T cell ratios (Figure 3L,M) was influenced by D‐mannose. Taken together, these data suggest that D‐mannose administration does not critically regulate the gut microbiome and circulatory T lymphocytes in treating T2D.

### 2.4 D‐Mannose Therapy Alleviates Hepatic Steatosis and Insulin Resistance

**FIGURE 4:**
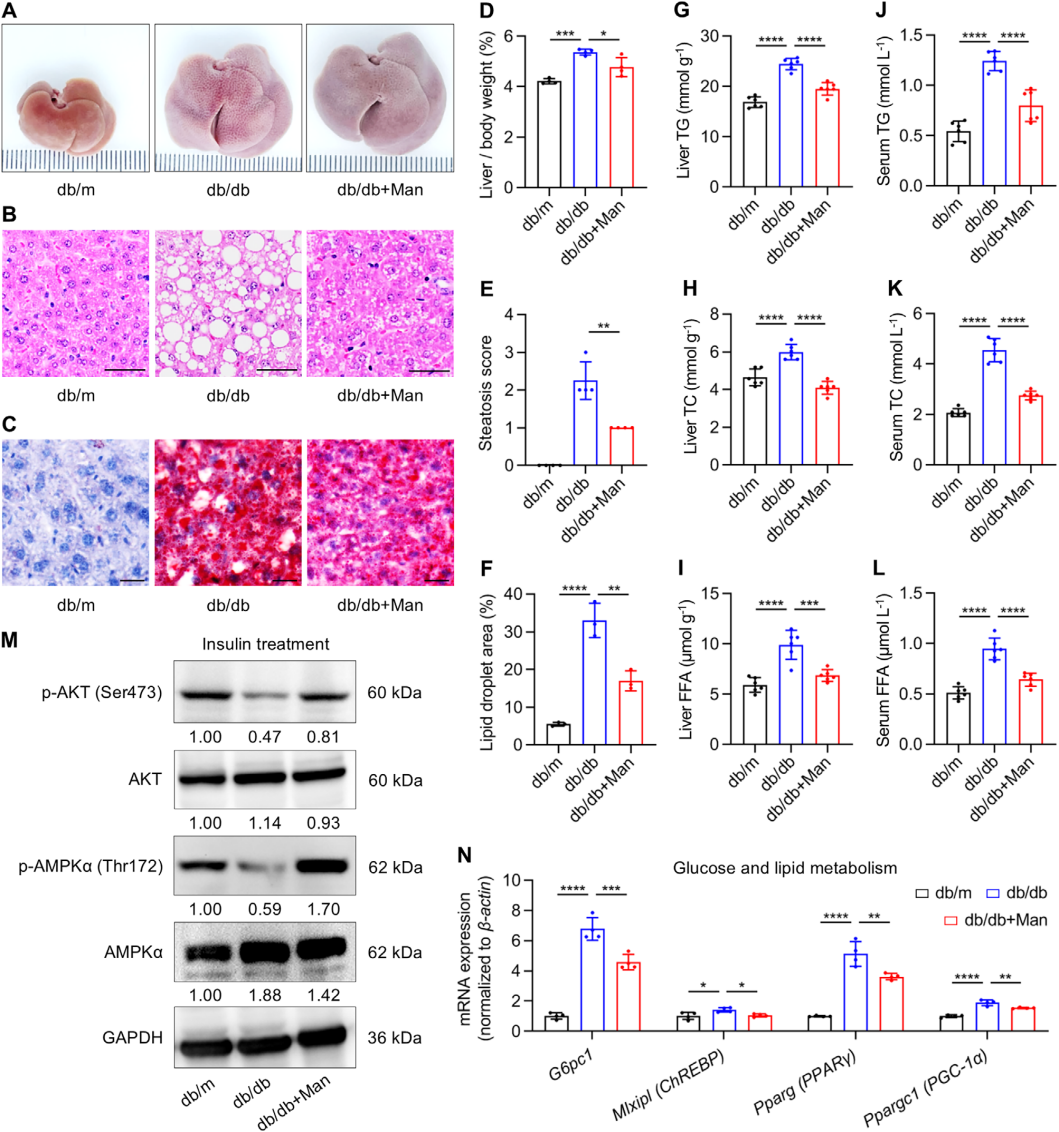
D‐mannose therapy alleviates hepatic steatosis and insulin resistance. (A) Gross view images of the liver. Man, mannose. (B) Hematoxylin and eosin (H&E) staining images showing the liver histology. Scale bars, 100 µm. (C) Oil red O staining images showing lipid droplets in the liver. Scale bars, 25 µm. (D) Ratio of liver weight over body weight, *n* = 4. (E) Quantification of hepatic steatosis in H&E staining images, *n* = 4. (F) Quantification of lipid droplet area percentages in oil red O staining images, *n* = 3. (G) Liver triglyceride (TG) contents analyzed by chemical tests, *n* = 6. (H) Liver total cholesterol (TC) contents analyzed by chemical tests, *n* = 6. (I) Liver free fatty acid (FFA) contents analyzed by chemical tests, *n* = 6. (J) Serum TG contents analyzed by chemical tests, *n* = 6. (K) Serum TC contents analyzed by chemical tests, *n* = 6. (L) Serum FFA contents analyzed by chemical tests, *n* = 6. (M) Western blot analysis of phosphorylation levels of AKT and adenosine 5′‐monophosphate (AMP)‐activated protein kinase alpha (AMPKα) in the liver after 1 IU kg^−1^ insulin treatment for 15 min. Grey values of bands were relative to those of the db/m group and normalized to the respective GAPDH band. (N) Quantitative real‐time polymerase chain reaction (qRT‐PCR) analysis of glucose and lipid metabolic gene expression in the liver, *n* = 4. Mean ± SD. *, *p* < 0.05; **, *p* < 0.01; ***, *p* < 0.001; ****, *p* < 0.0001. One‐way ANOVA with Turkey's post‐hoc test.

The liver is a major metabolic organ which pathologically undergoes metabolic dysfunction, develops fatty liver disease, reveals insulin resistance and contributes to T2D [4]. As liver is also the primary organ for circulatory mannose consumption (Figure 3A) [37], we next evaluated whether D‐mannose improved hepatic conditions in treating T2D. Gross analysis detected that the db/db liver had steatosis appearance, while D‐mannose administration benefited the liver status (Figure 4A). D‐mannose amelioration of the fatty liver in db/db mice was further confirmed at the histological level by hematoxylin and eosin (H&E) staining (Figure 3B), as well as oil red O (ORO) staining (Figure 3C), which revealed decreased lipid droplet area in the D‐mannose‐treated liver. Accordingly, D‐mannose reduced the liver/body weight ratio (Figure 4D) and alleviated hepatic steatosis with statistical significance in db/db mice (Figure 4E,F). Moreover, the elevated contents of triglyceride (TG), total cholesterol (TC), and free fatty acids (FFA) in the db/db liver were rescued by D‐mannose therapy (Figure 4G–I), which were further correlated with recovered hyperlipidemia (Figure 4J–L). We have also examined the hepatic insulin resistance by performing Western blot analysis after insulin injection. Data demonstrated that phosphorylation levels of protein kinase B (AKT) and adenosine 5′‐monophosphate (AMP)‐activated protein kinase alpha (AMPKα) were decreased in the db/db liver, which suggested impaired insulin sensitivity and were rescued by D‐mannose therapy (Figure 4M). Notably, D‐mannose administration also restored expression related to hepatic glucose output and lipid metabolism, including glucose‐6‐phosphatase (encoded by *Glucose‐6‐phosphatase catalytic subunit 1*, *G6pc1*), carbohydrate response element binding protein (ChREBP, encoded by *MLX interacting protein like*, *Mlxipl*), peroxisome proliferator‐activated receptor gamma (PPARγ, encoded by *Pparg*) and PPARγ coactivator‐1alpha (PGC‐1α, encoded by *Ppargc1*). Taken together, these results suggest that D‐mannose therapy alleviates hepatic steatosis and insulin resistance.

### 2.5 D‐Mannose Improves Hepatocyte Function Through Suppressing Macrophage EV Release

**FIGURE 5:**
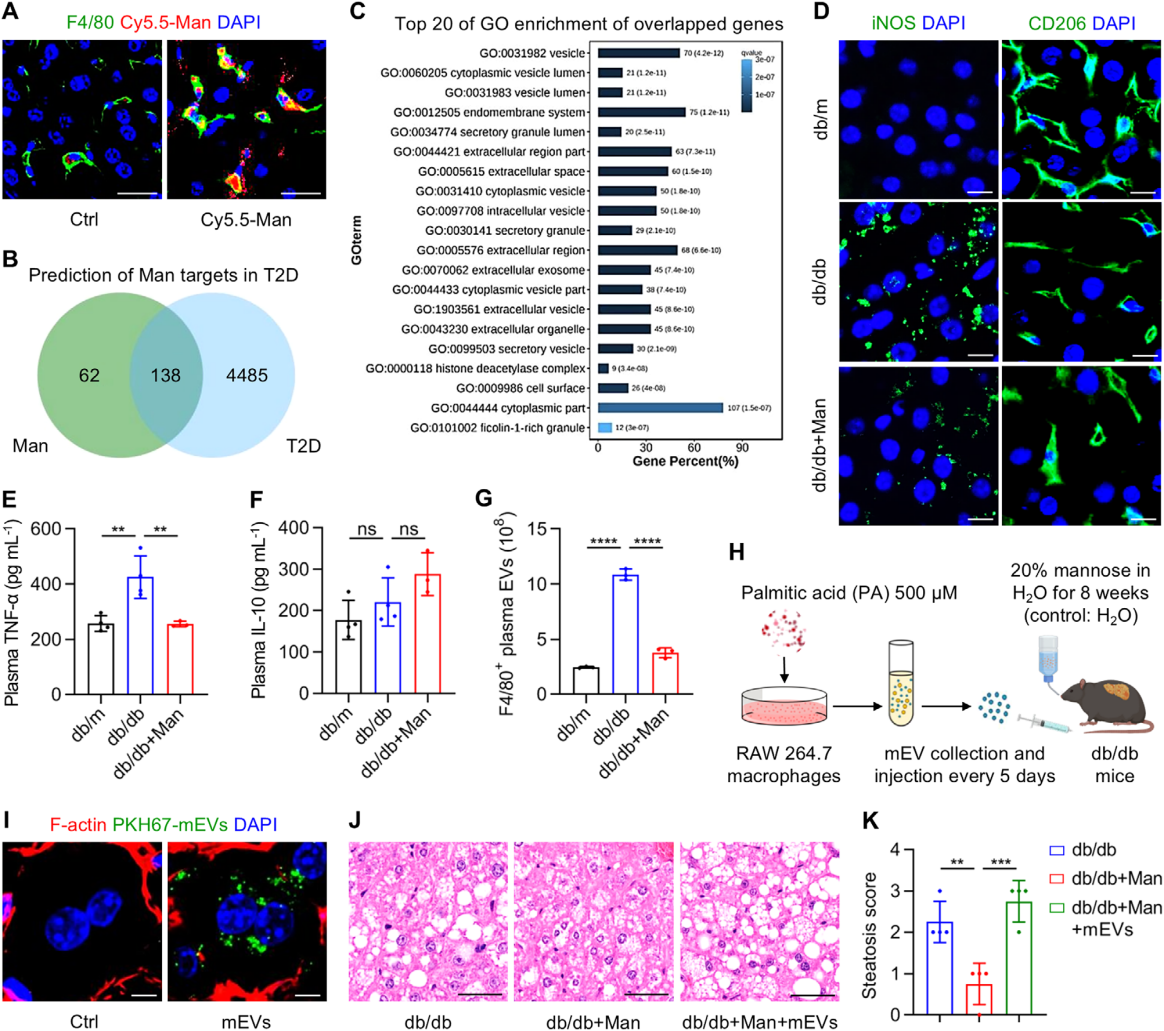
D‐mannose inhibits macrophage release of extracellular vesicles (EVs) for improving hepatocyte function. (A) Fluorescent images showing the internalization of Cy5.5‐labeled mannose (Man, red) by F4/80‐marked macrophages (green) in the liver, counteracted by DAPI (blue). Scale bars, 50 µm. (B) Prediction of potential mannose targets in type 2 diabetes (T2D) by network pharmacology. (C) Top 20 terms of Gene oncology (GO) enrichment analysis of overlapped genes of mannose and T2D. (D) Fluorescent images showing inducible nitric oxide synthase (iNOS) or CD206 (green) positive area in the liver, counteracted by DAPI (blue). Scale bars, 25 µm. (E) Enzyme‐linked immunosorbent assay (ELISA) of plasma TNF‐α levels, *n* = 3–4. (F) ELISA of plasma interleukin‐10 (IL‐10) levels, *n* = 3–4. (G) Quantification of F4/80^+^ macrophage‐produced EVs in the plasma by nanoparticle tracking analysis (NTA) combined with flow cytometric analysis, *n* = 3. (H) The diagram showing the experimental procedure of macrophage‐derived EV (mEV) collection and injection. PA, palmitic acid. (I) Fluorescent images showing the uptake of PKH67‐labeled mEVs (green) by F‐actin‐labeled hepatocytes (red) in the liver, counteracted by DAPI (blue). Scale bars, 5 µm. (J) Hematoxylin and eosin (H&E) staining images showing the liver histology. Scale bars, 100 µm. (K) Quantification of hepatic steatosis in H&E staining images, *n* = 4. Mean ± SD. **, *p* < 0.01; ***, *p* < 0.001; ****, *p* < 0.0001; ns, *p* > 0.05. One‐way ANOVA with Turkey's post‐hoc test.

Next, we deciphered how D‐mannose may improve hepatic steatosis. The mannose receptor (also termed CD206) is predominantly expressed on macrophages, among other cells, and modulates their polarization and inflammatory response [40]. Accordingly, we have performed immunofluorescent (IF) staining and discovered that F4/80‐marked liver macrophages were the main cells internalizing Cy5.5‐labeled mannose (Figure 5A). We have also adopted the network pharmacology method to predict molecular targets of D‐mannose in treating T2D, which showed 138 overlapped genes between D‐mannose and T2D (Figure 5B). Of these potential targets, gene oncology (GO) enrichment analysis revealed that most top‐enriched terms were related to EVs (Figure 5C), which was notable and surprising. Thus, we investigated that whether liver macrophages were regulated by D‐mannose therapy and that whether macrophage release of EVs was involved. Further IF staining of macrophage activation/polarization markers in the liver, inducible nitric oxide synthase [41] (iNOS, pro‐inflammatory) and CD206 per se (anti‐inflammatory) (Figure 5D), as well as enzyme‐linked immunosorbent assay (ELISA) of plasma TNF‐α and IL‐10 (anti‐inflammatory) concentrations (Figure 5E,F), exhibited that D‐mannose suppressed the pro‐inflammatory reaction of liver macrophages without affecting the anti‐inflammatory response. Particularly, db/db mice showed a notable characteristic of increased F4/80^+^ macrophage‐derived EV population in circulation, which was restored by D‐mannose therapy (Figure 5G and Figure S3B). These results suggest that paracrine effects of macrophages, especially EV release, are involved in D‐mannose therapy of T2D.

To confirm whether D‐mannose regulation of macrophage release of EVs was indeed critical to its therapeutic efficacy, we cultured the RAW264.7 mouse macrophage cell line, treated them with palmitic acid (PA), a saturated fatty acid to mimic the T2D environment [42], collected macrophage‐derived extracellular vesicles (mEVs) by differential centrifugation, and injected mEVs intermittently back into D‐mannose‐administered db/db mice (Figure 5H). The isolated mEVs demonstrated featured size distribution ranging from 50–500 nm peaked at 100–200 nm (Figure S4A), a typical cup‐shaped morphology (Figure S4B), expression of macrophage surface markers (Figure S4C) and representative EV proteins (Figure S4D) [43]. Biodistribution analysis of DiR‐labeled mEVs showed remarkable liver accumulation (Figure S3C), and fluorescent tracing of PKH67‐labeled mEVs in the recipient liver identified that almost all infused mEVs were detected within the F‐actin‐marked hepatocyte cell border, indicating uptake of mEVs by hepatocytes (Figure 5I). Accordingly, treatment of cultured hepatocytes with mEVs (Figure S4E) demonstrated that mEVs from the db/db macrophages inhibited glucose and fatty acid uptake while promoting glucose and lipid output of hepatocytes (Figure S4F–I), suggesting that macrophage pathological EVs may be a key factor in disordered diabetic liver metabolism. Confirmed as expected, replenishment of PA‐preconditioned mEVs in D‐mannose‐treated db/db mice blocked therapeutic effects of D‐mannose on hepatic steatosis, leaving pathological lipid droplet deposition despite D‐mannose administration (Figure 5J,K). Taken together, these findings suggest that D‐mannose improves hepatocyte function through suppressing macrophage EV release.

### 2.6 D‐Mannose Metabolism Suppresses CD36 Expression in Macrophages to Control EV Release

**FIGURE 6:**
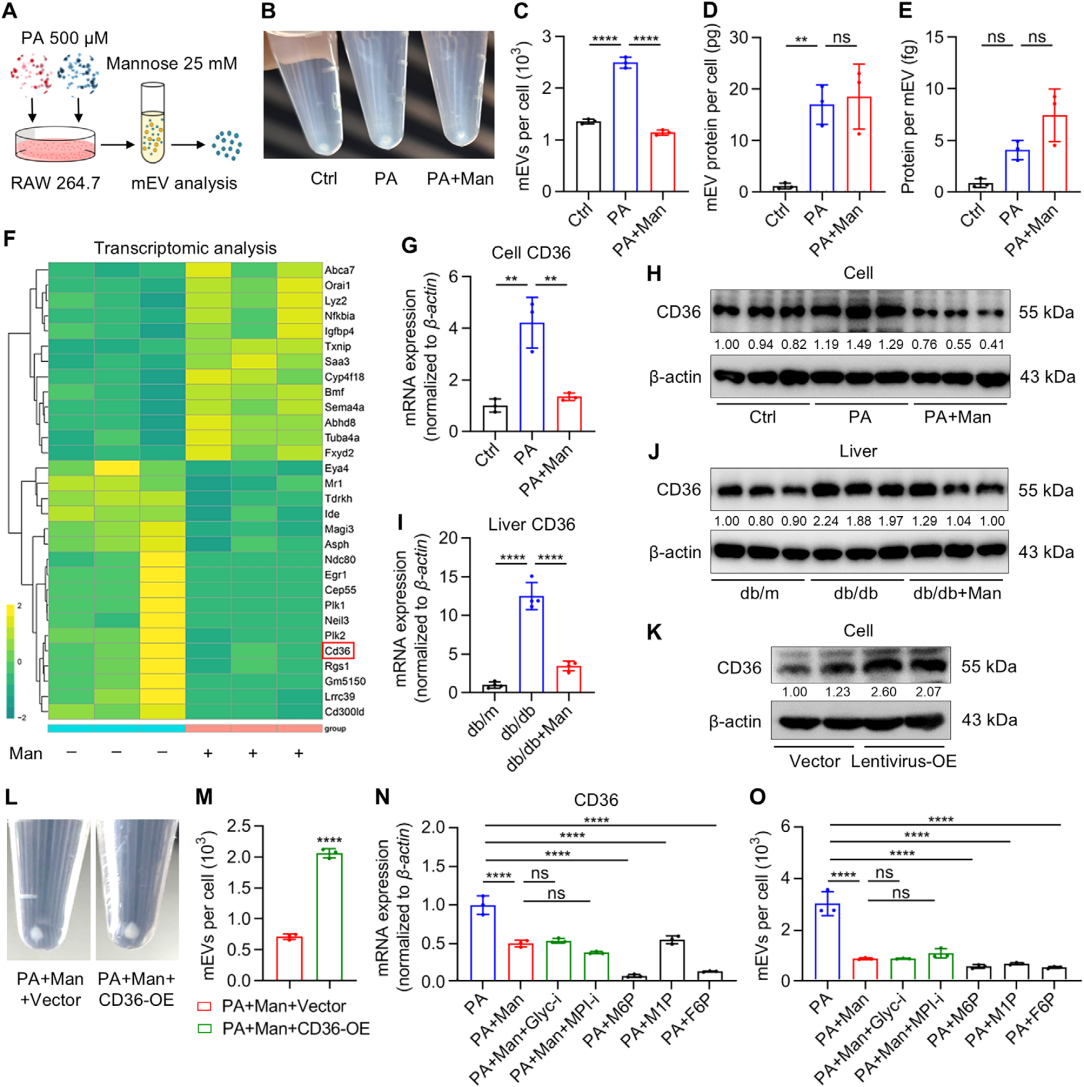
D‐mannose metabolism suppresses CD36 expression to control macrophage extracellular vesicle (EV) release. (A) The diagram showing palmitic acid (PA) and mannose (Man) treatment of macrophages for analyzing macrophage‐derived EVs (mEVs). (B) The gross view image of collected mEVs in 1.5‐mL tubes from one 6‐well macrophages. (C) Quantification of mEVs by nanoparticle tracking analysis (NTA), *n* = 3. (D) Quantification of mEV protein content by BCA assay, *n* = 3. (E) Quantification of protein content per mEV, *n* = 3. (F) mRNA sequencing analysis of macrophages with or without mannose treatment. (G) Quantitative real‐time polymerase chain reaction (qRT‐PCR) analysis of CD36 expression in macrophages, *n* = 3. (H) Western blot analysis of CD36 expression in macrophages. Grey values of bands were relative to the far left Ctrl band and normalized to the respective β‐actin band. (I) qRT‐PCR analysis of CD36 expression in the liver, *n* = 4. (J) Western blot analysis of CD36 expression in the liver. Grey values of bands were relative to the far left db/m band and normalized to the respective β‐actin band. (K) Western blot analysis of CD36 expression in macrophages after transfection of CD36 over expression (OE) lentivirus or its vector. Grey values of bands were relative to the far left Vector band and normalized to the respective β‐actin band. (L) Gross view images of collected mEVs in 1.5‐mL tubes from one 6‐well macrophages. (M) Quantification of mEVs by NTA, *n* = 3. (N) qRT‐PCR analysis of CD36 expression in macrophages, *n* = 3. Glyc‐i, inhibitor of protein *N*‐glycosylation; MPI‐i, inhibitor of mannose‐6‐phosphate isomerase; M6P, mannose‐6‐phosphate; M1P, mannose‐1‐phosphate; F6P, fructose‐6‐phosphate. (O) Quantification of mEVs by NTA, *n* = 3. Mean ± SD. **, *p* < 0.01; ****, *p* < 0.0001; ns, *p* > 0.05. Two‐tailed unpaired Student's *t* test (M) or One‐way ANOVA with Turkey's post‐hoc test (C–E, G, I, N, and O).

Next, we dissected how D‐mannose may regulate macrophage release of EVs. RAW 264.7 mouse macrophages were treated with PA and D‐mannose according to the determined dosage (Figure 6A and Figure S5A). We confirmed that the number of mEVs released, rather than their protein content, was promoted by PA and was restored by D‐mannose (Figure 6B–E). Besides, D‐mannose treatment still suppressed PA‐triggered pro‐inflammatory activation of macrophages in vitro (Figure S5B,C). To explore the potential molecule(s) mediating effects of D‐mannose, we performed next‐generation RNA‐sequencing analysis on macrophages (Table S2), and the transcriptome data suggested multiple genes modulated, of which CD36, a recently reported regulator of EV release and fatty acid uptake, was involved (Figure 6F) [44]. qRT‐PCR analysis of CD36 expression in cultured macrophages confirmed its upregulation after PA treatment, which was suppressed by D‐mannose (Figure 6G). Protein expression of CD36 examined by Western blot analysis supported the suppressive effects of D‐mannose against PA (Figure 6H). We have also evaluated CD36 expression in vivo in liver samples, and data showed that the db/db liver had increased CD36 expression compared to db/m, which was inhibited by D‐mannose therapy (Figure 6I,J). These results suggest macrophage CD36 as a candidate target for mediating D‐mannose effects.

To prove that whether CD36 indeed contributed to D‐mannose regulation of EV release in macrophages, we performed lentivirus‐based gene over expression (OE) in macrophages, and the CD36‐OE efficacy was confirmed by Western blot analysis (Figure 6K). Importantly, we revealed that CD36‐OE reversed suppressive effects of D‐mannose on PA‐induced mEV release (Figure 6L,M), indicating that CD36 was the key to mediating D‐mannose effects. To further investigate how D‐mannose may regulate CD36 expression in macrophages, we applied chemical inhibitors of MPI and protein N‐glycosylation to respectively block each of the two metabolic cascades of D‐mannose [25]. We have also tested metabolites of D‐mannose along the cascades, mannose‐6‐phosphate (M6P), mannose‐1‐phosphate (M1P), and fructose‐6‐phosphate (F6P), by treating PA‐conditioned macrophages instead of D‐mannose. Intriguingly, we discovered that neither protein N‐glycosylation nor MPI inhibition was able to counteract D‐mannose to suppress CD36 expression and mEV release under PA treatment, whereas each of the M6P, M1P and F6P was enough to replicate D‐mannose effects (Figure 6N,O). These above results indicate that D‐mannose potently controls macrophage EV release by robust suppression of the CD36 gene expression through itself and its metabolic processes.

### 2.7 D‐Mannose Retards Diabetic Bone Loss Based on Suppression of Macrophage EV Release

**FIGURE 7:**
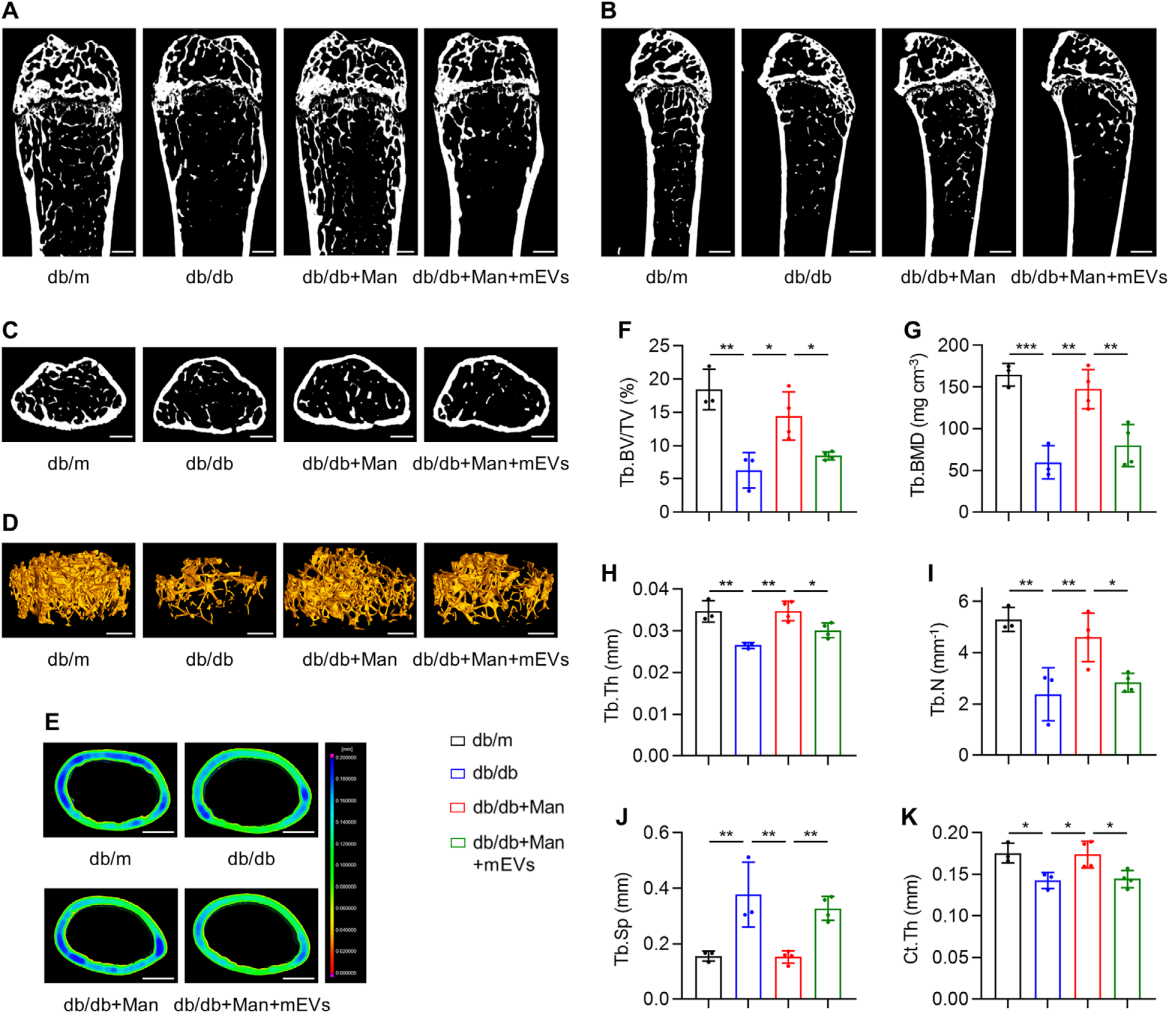
D‐mannose retards diabetic bone loss based on suppression of macrophage extracellular vesicle (EV) release. (A) Micro‐CT images show the coronal sectional view of mouse distal femora. Bars: 500 µm. Man, mannose; mEVs, macrophage‐derived EVs. (B) Micro‐CT images show the sagittal sectional view of mouse distal femora. Bars: 500 µm. (C) Micro‐CT images show the horizontal sectional view of mouse distal femora. Bars: 500 µm. (D) Micro‐CT three‐dimensional reconstruction images show the trabecular bone mass of mouse distal femora. Bars: 500 µm. (E) Micro‐CT images show the cortical bone of mouse distal femora. Bars: 500 µm. (F) The parameter of trabecular bone volume over tissue volume (Tb.BV/TV), *n* = 3–4. (G) The parameter of trabecular bone mineral density (Tb.BMD), *n* = 3–4. (H) The parameter of trabecular thickness (Tb.Th) of trabecular bone mass, *n* = 3–4. (I) The parameter of trabecular number (Tb.N) of trabecular bone mass, *n* = 3–4. (J) The parameter of trabecular separation (Tb.Sp) of trabecular bone mass, *n* = 3–4. (K) The parameter of cortical thickness (Ct.Th) of cortical bone mass, *n* = 3–4. Mean ± SD. *, *p* < 0.05; **, *p* < 0.01; ***, *p* < 0.001. One‐way ANOVA with Turkey's post‐hoc test.

T2D has been reported to lead to various complications affecting multiple organ systems, and one significant complication that often arises in individuals with T2D is related to bone health [3, 5]. Thus, finally, we investigated that whether D‐mannose administration improved multi‐organ conditions in T2D by examining bone status, and that whether the effect was based on macrophage EV regulation. Expectedly, we discovered that db/db mice developed remarkable trabecular bone loss during the experimental period, as shown by micro‐computed tomography (micro‐CT) images demonstrating the distal epiphysis and metaphysis of femora (Figure 7A–C). Three‐dimensional reconstruction of the trabecular bone confirmed the changes in db/db mice, with further a diminished thickness of the cortical bone in the midshaft region (Figure 7D,E). Importantly, despite limited biodistribution of exogenous D‐mannose in the skeleton (Figure 3A), oral administration of D‐mannose substantially retarded both the trabecular and the cortical bone loss in db/db mice (Figure 7A–E). Furthermore, these effects were suppressed by replenishment of PA‐induced pathological mEVs (Figure 7A–E). Statistical analyses of corresponding trabecular and cortical bone parameters verified the micro‐CT observations, showing altered bone micro‐architecture in db/db mice rescued by D‐mannose therapy, which was counteracted by mEV replenishment (Figure 7F–K). Collectively, these results indicate that D‐mannose retards diabetic bone loss based on suppression of macrophage EV release.

**FIGURE 8:**
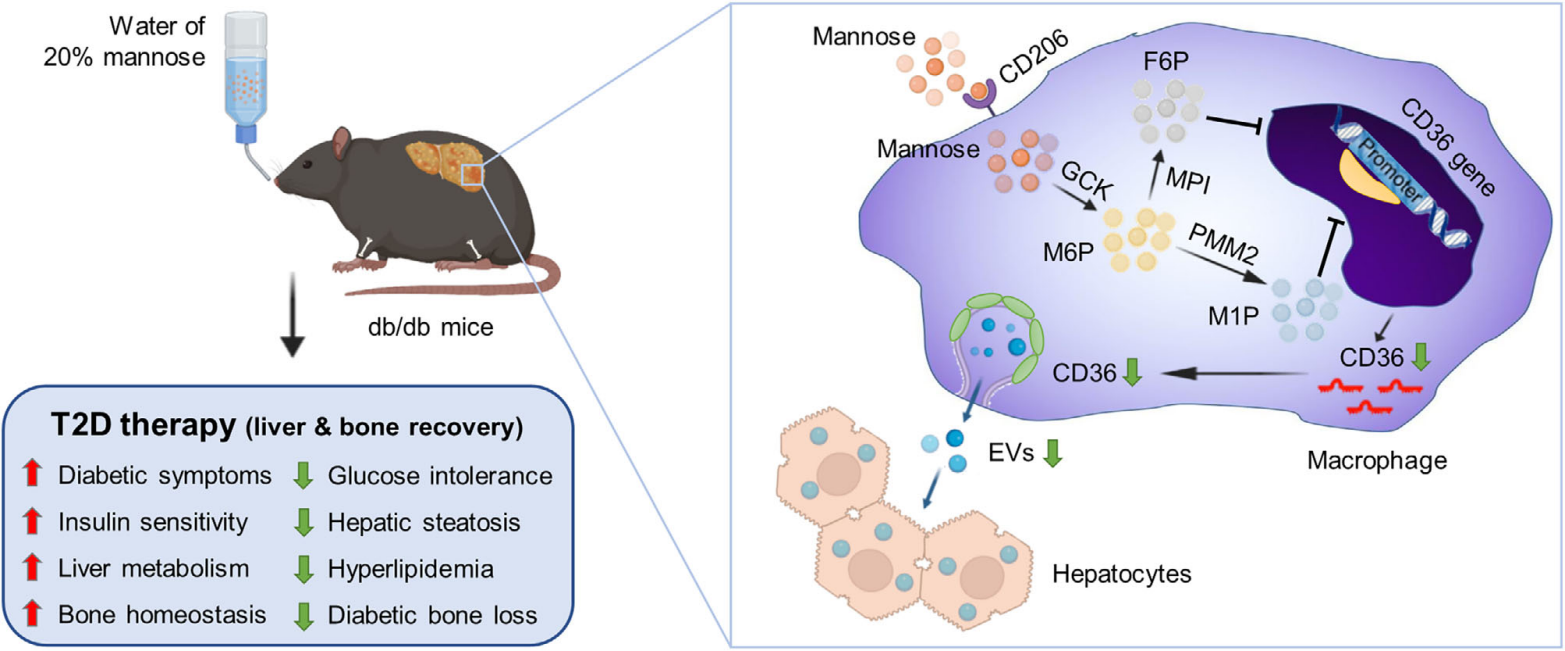
Working model of oral administration of D‐mannose ameliorating type 2 diabetes (T2D) and multi‐organ deteriorations in db/db mice. D‐mannose is dissolved in drinking water at 20 g per 100 mL for treating db/db mice for 8 weeks. D‐mannose is internalized by macrophages via the CD206 receptor and is metabolized through a series of enzymes. Mannose with its metabolites is capable of suppressing CD36 gene expression, which subsequently inhibits release of hepatocyte‐regulating extracellular vesicles (EVs). Accordingly, multiple symptoms of T2D are ameliorated, particularly including hepatic steatosis and bone loss. GCK, glucokinase; PMM2, phosphomannomutase 2; MPI, mannose‐6‐phosphate isomerase; M6P, mannose‐6‐phosphate; M1P, mannose‐1‐phosphate; F6P, fructose‐6‐phosphate.

Taken together, the main findings of this study uncover an effective and potential T2D therapeutic by oral delivery of D‐mannose, which rescued both hepatic steatosis and diabetic bone loss through suppressing macrophage release of EVs based on metabolic control of CD36 expression (Figure 8). Our findings suggest that naturally existed sugars can regulate intercellular EV messages and shed light on a potential T2D approach with multi‐organ benefits.

## 3 Discussion

Dysregulation of the mannose metabolism in obese individuals has been previously documented to underlie a shift in the utilization of carbohydrate substrates in the liver, but the hepatic expression of genes responsible for mannose processing was detected with complicated results [21]. Lee et al., have reported that the hepatic expression of *GCK*, the main hexokinase in the liver to converts D‐mannose to M6P, was upregulated in obese subjects with co‐upregulated *PMM1* and *Guanosine diphosphate (GDP)‐mannose pyrophosphorylase A* (*GMPPA*)/*GMPPB*, which respectively converts M6P to M1P and M1P to GDP‐mannose for the downstream N‐glycan metabolism [21]. They have also reported downregulated *Hexokinase 1* (*HK1*) and *HK2* in the liver of obese people and proposed a causal relationship between hepatic changes of these genes with the increased plasma level of mannose, hypothesizing a reduced capability of mannose consumption despite contradictory data among the detected gene expression [21]. In the present study, we perform further analysis of the hepatic expression of genes related to mannose metabolism in db/db mice, which not only include mannose processing enzymes but also mannose synthesizing enzymes in the gluconeogenesis cascade. Our findings show upregulated expression of mannose synthesizing enzymes, *Pck1* and *Fbp1*, and confirm upregulation of *Gck* in the liver of obese subjects, which suggest increased endogenous mannose production with sustained capability of mannose consumption in T2D. Our results thus provide a progressive perspective explaining the high mannose level in T2D as only a side‐effect of elevated gluconeogenesis and serve as one theoretical basis for the organism to potently utilize exogenously administered mannose for therapy. These results also suggest that an increase in mannose might be a metabolic regulatory concomitant response to diabetes, rather than directly contributing to the condition.

D‐mannose is easily obtained naturally to made ready‐to‐use dietary powders for food, healthcare and clinical usage [45]. In human, long‐term efficacy and safety of oral D‐mannose administration have been recognized in treating MPI‐congenital disorder of glycosylation (MPI‐CDG) and recurrent urinary tract infection (UTI) [26, 27]. In mice, initial important works from the Chen group have revealed D‐mannose as an inducer of Foxp3^+^ regulatory T cells (Treg cells) by promoting transforming growth factor‐beta (TGF‐β) based on increased fatty acid oxidation [28]. Accordingly, drinking‐water supplementation of D‐mannose has been applied to improve autoimmune T1D and airway inflammation [28]. Immunoregulatory function of D‐mannose has been later documented by Torretta et al., to involve suppression of macrophage production of IL‐1β by accumulated intracellular M6P impairing the glucose utilization, which contributed to alleviation of lipopolysaccharide (LPS)‐induced endotoxemia and dextran sulfate sodium (DSS)‐induced colitis in mice [29]. Sharma et al., have additionally reported that less energy harvest by the gut microbiota partially contributes to D‐mannose‐prevented dietary obesity in mice, during which the effects were closely related to the timing and duration of initial mannose administration [30]. In this study, we surprisingly discover that providing D‐mannose in early life does not ameliorate the obese phenotype in db/db mice, nor affect the gut microbiome diversity. Furthermore, D‐mannose administration in our study does not influence the peripheral blood T lymphocyte percentages, nor directly target hepatocytes in vivo, as exhibited by fluorescent tracing images. However, drinking‐water supplementation of D‐mannose indeed rescues the NAFLD phenotype, improves hepatic insulin sensitivity and alleviates T2D in db/db mice, which are based on macrophage regulation but notably, independent of bioenergetic modulatory effects. Importantly, through regulating macrophage EV release, D‐mannose administration also reveals extra‐hepatic benefits and significantly retards diabetic bone loss. Therefore, our findings add to the current mechanistic understanding of D‐mannose effects, indicating gene transcription regulation by mannose and its metabolites is necessary and important. How D‐mannose and its metabolites suppress CD36 expression in macrophages will require further studies.

The metabolic homeostasis depends on the complex, multi‐directional crosstalk between local and distant cells, which becomes dysregulated in metabolic diseases [46]. Accumulating evidence has supported a role of EVs in obesity‐associated T2D metabolic disturbance, particularly the regional and systemic inflammation characteristics of macrophages related to adipose and hepatic stress [47, 48]. The Olefsky group has established that in obese mice, pro‐inflammatory adipose tissue macrophages (ATMs) secrete miRNA‐155‐containing exosomes, endosome‐originated EVs [49, 50], to reduce insulin sensitivity in target organs, including the liver [11]. They have further documented that the anti‐inflammatory M2‐polarized bone marrow‐derived macrophages (BMDMs) secrete miRNA‐690‐transferring exosomes to rescue insulin resistance in remote organs in HFD mice [51]. We have previously reported that infusion of liver macrophage‐targeting EVs ameliorates the pro‐inflammatory niche and rescues hepatic steatosis and T2D under dietary obesity in mice [52]. These findings collectively suggest that macrophage‐based modulation of release or composition of endogenous EVs would benefit the metabolic status despite obesity, but feasible methods are limited. In the present study, we provide a readily accessible pharmaceutical approach to control macrophage release of pathological EVs for T2D treatment, which will thus have immense translational value. Also notably, glycosylation participates in biogenesis, release and distribution of EVs, and surface D‐mannose modification affects the fate and uptake of exogenously delivered exosomes [32, 53]. Moreover, well identified as a fatty acid transporter and a plasma membrane glycoprotein, CD36 is reported to regulate the ceramide formation in the caveolae to ensure release of fatty acid‐containing exosome‐like EVs, which is heavily modified at the post‐translation level by N‐linked glycosylation to mediate its trafficking to the plasma membrane and function [44, 54]. Our results reveal that CD36 expression is regulated by both D‐mannose and its multiple metabolites, suggesting an effective and stable mechanism of function. Although we focus on gene transcription regulation in this study, whether D‐mannose via the glycosylation process influences CD36 might provide in‐depth mechanistic understanding of EV release and novel therapeutic targets of T2D in future works.

Macrophages and CD36 are both therapeutically exploited targets in immunotherapy or nanomedicines [55, 56], while targeting endogenous EVs is insufficiently investigated. Existing literature indicates that the M1 pro‐inflammatory polarization of macrophages is associated with altered cargoes of EVs they release, such as the above‐mentioned miR‐155, but macrophage polarization might have limited influence on the quantity of EVs released [11, 57]. In this study, although that D‐mannose effects on macrophage M1 polarization and EV release are concomitant, there is also no direct evidence on potential overlapping pathways linking these two effects. Nevertheless, CD36 has been documented to promote inflammatory responses of macrophages under PA challenge [58], suggesting possible interrelationships of the two regulatory arms in a lipid‐rich microenvironment. Furthermore, a subpopulation of CD206^hi^Endothelial cell adhesion molecule (ESAM)^+^ liver macrophages has been recently revealed to be metabolically responsive through the expression of CD36, which promotes oxidative stress associated with diet‐induced obesity and hepatic steatosis [59]. Importantly, targeted silencing of *Cd36* expression in liver macrophages improves glucose tolerance and ameliorates hyperglycemia in HFD‐fed mice, confirming the translational implication of CD36 [59]. Another noticeable point is that D‐mannose does not rescue the obese phenotype of db/db mice, despite amelioration of T2D symptoms. Although that obesity and T2D share pathophysiological mechanisms, these two diseases are not always coupled in treatments [60, 61]. One possible explanation is in the central neural regulation of body weight, which is critically affected by leptin functioning on the brain [62]. Therefore, D‐mannose is effective in alleviating dietary obesity [30], but fails to control overweight in db/db mice, which surely have central deficiency of leptin receptor. This evidence suggests that D‐mannose might mainly exert peripheral effects directly on target organs.

## 4 Conclusions

In summary, we uncover that drinking‐water supplementation of D‐mannose serves as a candidate T2D therapeutic, which rescued hepatic steatosis and diabetic bone loss through suppressing macrophage release of EVs based on metabolic control of CD36 expression. Our findings pave an avenue for novel translational pharmaceutical strategies of T2D with its multi‐organ deteriorations.

## Author Contributions

**Sha Zhang**: data curation, formal analysis, investigation, validation, visualization, writing – original draft. **Kai Zhang**: data curation, formal analysis, investigation, validation, visualization, visualization, writing – original draft. **Chen‐Xi Zheng**: Conceptualization, data curation, formal analysis, funding acquisition, investigation, supervision, validation, visualization, writing – original draft, writing – review and editing. **Ying‐Feng Gao**: data curation, formal analysis, investigation, validation, visualization, writing – original draft. **Guo‐Rong Deng**: methodology, software. **Xu Zhang**: methodology, software. **Yuan Yuan**: methodology, software. **Ting Jia**: methodology, software. **Si‐Yuan Tang**: methodology, software. **Guang‐Xiang He**: methodology, software. **Zhen Gong**: methodology, software. **Na Zhao**: project administration, resources. **Bo Ma**: project administration, resources. **Hua Tian**: project administration, resources. **Hong Zhang**: project administration, resources. **Zhe Li**: project administration, resources. **Yong‐Chang Di‐Wu**: project administration, resources. **Yi‐Han Liu**: project administration, resources. **Liang Kong**: project administration, resources. **Jing Ma**: Conceptualization, funding acquisition, supervision, writing – review and editing. **Yan Jin**: Conceptualization, funding acquisition, supervision, writing – review and editing. **Bing‐Dong Sui**: Conceptualization, funding acquisition, supervision, writing – review and editing. All authors had accessed and verified the underlying data and read and approved the final version of the manuscript.

## Conflicts of Interest

The authors declare no conflicts of interest.

## Supporting information




**Supplementary File 1**: exp270083‐sup‐0001 SuppMat.pdf

**Supplementary File 2**: exp270083‐sup‐0002 TableS1.xlsx

**Supplementary File 3**: exp270083‐sup‐0003 TableS2.xlsx

**Supplementary File 4**: exp270083‐sup‐0004 TableS3.xlsx

**Supplementary File 5**: exp270083‐sup‐0005 TableS4.docx

## Data Availability

The data that support the findings of this study are available from the corresponding authors upon reasonable request.
