# Supplementary material for: D‐Mannose Alleviates Type 2 Diabetes and Rescues Multi‐Organ Deteriorations by Controlling Release of Pathological Extracellular Vesicles
Source: Exploration (Beijing). 2025 Aug 25;5(5):20240133. doi: 10.1002/EXP.20240133 (PMC12561340; doi:10.1002/EXP.20240133)
Supplement: Supplementary file 1 — exp270083‐sup‐0001 SuppMat.pdf [file EXP2-5-20240133-s001.pdf]

Materials and Methods

Mice

BKS.Cg-*Dock7*^{m} +/+ *Lepr*^{db}/J mice (strain NO. 000642) were purchased from the Jackson Laboratory, USA. Non-obese and non-diabetic heterozygotes from the colony (denoted as db/m) were used as the control and for breeding of the obese and diabetic *Lepr*^{db}/+ *Lepr*^{db} (denoted as db/db) homozygotes. Mice in each group were used at n=3-6 single animals according to the experimental design with no criteria set for exclusion of animals or data points. Mice were randomized allocated based on the randomized number table with no confounders controlled. The researchers were blind to the group allocation throughout the experiments. Male mice were used to exclude the potential side effects caused by estrogen. Male mice were used from 5-week old to 13-week old, which were housed in pathogen-free conditions, maintained on a standard 12-h light-dark cycle, and received normal chow diet and water *ad libitum*. The study did not set humane endpoints or report any adverse events with no protocols prepared before the study. All animal experiments were performed in compliance with the relevant laws and ethical regulations, following the Guidelines of Intramural Animal Use and Care Committees of The Fourth Military Medical University, approved by the Ethics Committee of The Fourth Military Medical University (No. kq-2023-045), and following the ARRIVE guidelines.

Cell lines

The RAW 264.7 mouse macrophage cell line was obtained from the American Type Culture Collection (TIB-71; ATCC, USA). Cells were cultured in Dulbecco's Modified Eagle Medium with 1 g L^{-1} D-glucose (low-glucose DMEM; Invitrogen, USA) supplemented with 10%

fetal bovine serum (FBS; ExCell Bio, China), 2 mM L-glutamine (Invitrogen, USA) and 1% penicillin/streptomycin (Invitrogen, USA) and incubated at 37°C under 5% CO_2.

Primary cell cultures

For culture of primary macrophages from the bone marrow (BMDMs), long bones of mice were harvested and bone marrow cavities were flushed with phosphate-buffered saline (PBS; Invitrogen, USA), which was then passed through a cell strainer and subjected to red blood cell lysis (Solarbio, China). Freshly isolated cells were cultured in low-glucose DMEM (Invitrogen, USA) supplemented with 10% FBS (ExCell Bio, China), 2 mM L-glutamine (Invitrogen, USA), 1% penicillin/streptomycin (Invitrogen, USA) and 20 ng ml^{-1} recombinant mouse macrophage-colony stimulating factor (M-CSF; PeproTech, USA). After induction for 7 days, mature BMDMs were collected and used for collection of macrophage-derived extracellular vesicles (mEVs).

Isolation of primary mouse hepatocytes was performed by perfusion *via* the portal vein. Briefly, mouse liver was perfused *via* catheterization of the portal vein using a 24G needle catheter (BD, USA) and a mini-pump machine (Thermo Fisher Scientific, USA) under general anesthesia. The liver was perfused firstly with 10 ml Hank's balanced salt solution (HBSS) (Invitrogen, USA) to remove blood followed by 20 ml HBSS supplemented with 1 mM ethylene glycolbis(aminoethylether)-tetra-acetic acid (EGTA) (Sigma-Aldrich, USA) to remove the endogenous calcium. Then the liver was perfused with 20 ml HBSS supplemented with 5 mM calcium chloride ($CaCl_2$) (Sigma-Aldrich, USA) and 40 μg ml^{-1} liberase TM (Sigma-Aldrich, USA) for digestion. All the solutions were kept at 37ºC in a water bath. After digestion, the liver was dissected and washed in ice-chilled HBSS, and cells were teased out into DMEM with 4.5

g L^{-1} D-glucose (high-glucose DMEM; Invitrogen, USA) supplemented with 10% FBS (Sigma-Aldrich, USA) and 1% penicillin/streptomycin (Invitrogen, USA). Hepatocytes were then prepared by centrifugation at 50 g for 5 min at 4ºC, filtered through 70 μm nylon strainers, purified by a 49% Percoll solution (Sigma-Aldrich, USA), and resuspended in William's E Medium (WEM) with GlutaMAX™ (Invitrogen, USA) containing 10% FBS (Sigma-Aldrich, USA), 10 nM dexamethasone (Invitrogen, USA) and 1% penicillin/streptomycin (Invitrogen, USA). Hepatocytes were then seeded onto collagen-coated plates (Corning, USA) or coverslips (Electron Microscopy Sciences, USA) and incubated at 37ºC in a humidified atmosphere of 5% CO_2 overnight for attachment.

Chemical treatments

Drinking-water supplementation of D-mannose was performed by dissolving 20 g D-mannose (Shanghai Yuanye Bio-Technology, China) in 100 mL distilled water (20% or 0.2 g mL^{-1}, equal to 1.1 mol L^{-1}), and unsupplemented control water was given as the control. *In vitro* treatment of D-mannose was used at the concentration of 25 mM for 48 h for most experiments, and at different concentrations for the dose-effect experiment. To investigate the metabolic effect of D-mannose, Tunicamycin (MedChemExpress, China) was used to block protein N-glycosylation at 100 ng mL^{-1} for 48 h, and MLS0315771 (MedChemExpress, China) was applied to suppress mannose phosphate isomerase (MPI) at 5 mM for 48 h. Furthermore, to test the effects of D-mannose metabolites, mannose-6-phosphate (M6P) (Aladdin, China) was used at 25 mM (equal to the D-mannose concentration) for 48 h, mannose-1-phosphate (M1P) (Aladdin, China) was used at 1.25 mM (5% of the D-mannose dose) for 48 h, and fructose-6-phosphate (F6P) (Yingxinbio, China) was used at 23.75 mM (95% of the

D-mannose dose) for 48 h. Palmitic acid (PA) (Kunchuang Biotechnology, China) was added at 500 μM for 24 h.

CD36 overexpression

The overexpression of CD36 was performed using the lentivirus-based vector by Hanbio, China, with the vector being used as the negative control. After transfection at a multiplicity of infection (MOI) of 50, all RAW264.7 cells were treated with puromycin (Solarbio, China) at a concentration of 10 μg mL^{-1} for 10 days. The lentivirus transfection efficacy was validated by fluorescent imaging, quantitative real-time polymerase chain reaction (qRT-PCR) and Western blot analysis.

Isolation, labeling and treatment of mEVs

Extracellular vesicles (EVs) were isolated from cultured macrophages based on our established protocol. Briefly, cells were cultured in complete medium containing EV-depleted FBS for 48 h. EV-depleted FBS was obtained by ultracentrifugation at 100,000 g for 18 h. The culture supernatant was collected and subsequently centrifuged at 800 g for 10 min. The supernatant was further collected and centrifuged at 16,500 g for 30 min at 4ºC to obtain mEVs, which were then washed with filtered PBS. mEV pellets collected from each six-well were photographed, and quantification of mEVs was performed using the BCA method (Beyotime, China) for protein amounts. The lipophilic dye PKH67 (Sigma-Aldrich, USA) was used to label mEVs according to the manufacturer's instructions. In specific, after PKH67 staining for 5 min, mEV suspension in PBS was added by an equal volume of EV-depleted FBS and incubated for 1 min to allow binding of excess PKH67 dye. mEVs were then collected *via* centrifugation and washed with PBS to get rid of unbound PKH67. The

supernatant was used as the negative control, and the mEV pellets were resuspended for usage. The DiR dye (Yeasen, China) was used to label mEVs for biodistribution analysis according to the manufacturer's instructions. For *in vitro* treatment, mEVs were dissolved in PBS and added to the culture medium at a protein concentration of 20 μg mL^{-1}. For *in vivo* treatment, mEVs were dissolved in PBS and were infused *via* the caudal vein into recipient mice at 200 μg on the basis of protein measurement every 5 days during the experimental period.

Gross analysis and blood glucose quantification

Mice were recorded for body weight and water and food intake every 3 days. Cage bedding was photographed every 5 days. The concentrations of non-fasting random blood glucose were measured every 3 days throughout the experiments, and the concentrations of fasting blood glucose were measured after fasting for 6 h. Blood glucose was quantified using an ACCU-CKEK glucometer (Roche, Germany) following tail vein-puncture of whole blood sampling. The concentrations of glycated hemoglobin A1c (HbA1c) were determined using the A1CNow Self Check system (Sinocare, China). All mice were euthanasia at the end of experiments, photographed for gross view images, collected for organs and quantified for organ weights. Fat mass, lean mass and body fluid were determined using the minispec LF90 Whole Body Composition Analyzer (Bruker, Germany) before euthanasia.

Intraperitoneal glucose and insulin tolerance test (IPGTT and IPITT) assays

IPGTT and IPITT were performed as the primary outcome measures. For IPGTT, mice were fasted for 20 h and intraperitoneally injected with D-glucose (Oubokai, China) at 1.5 g kg^{-1}. For IPITT, mice were fasted for 6 h and intraperitoneally injected with recombinant

human insulin (Novo Nordisk, Denmark) at 2 IU kg^{-1}. Blood glucose levels were measured at 0, 15, 30, 60, 90 and 120 min after D-glucose or insulin administration.

Lipid content measurement

For hepatic lysate examination, at sacrifice, liver tissues were dissected at approximately 100 mg and homogenized in 9-fold volume of ethanol on ice. The lysates were centrifuged at 2,500 g for 10 min at 4ºC, and the supernatant was collected. For serum examination, at sacrifice, whole peripheral blood was extracted from the mouse retro-orbital venous plexus, and serum was isolated by centrifugation at 3,000 g for 15 min at 4ºC. Triglyceride (TG), total cholesterol (TC) and free fatty acids (FFA) levels were measured by the commercial kits according to the manufacturer's instructions (Nanjing Jiancheng Biology Engineering Institute, China).

Enzyme-linked immunosorbent assay (ELISA)

Plasma was isolated from extracted whole peripheral blood by adding heparin solution (STEMCELL Technologies, USA) followed by centrifugation at 1,300 g for 15 min at 4ºC. Concentrations of tumor necrosis factor-alpha (TNF-α) and interleukin-10 (IL-10) were determined using commercial kits (Fankewei, China) according to the manufacturer's instructions.

Peripheral blood T-cell analysis

Whole peripheral blood was extracted from the mouse retro-orbital venous plexus with anti-coagulation, and cells were isolated by centrifugation at 500 g for 5 min at 4ºC followed by being treated with a red blood lysis buffer (Beckman Coulter, USA). After washing with PBS, peripheral blood mononucleated cells (PBMNCs) were collected by centrifugation at 500 g for

5 min at 4ºC. PBMNCs were then stained with fluorescence-conjugated antibodies for CD3, CD4 and CD8 (all from BioLegend, USA) at 1:100 for 30 min at 4ºC in dark, washed and examined by the ACEA NovoCyte flow cytometer (Agilent, USA).

Flow cytometric analysis

For plasma EVs, collected plasma was further centrifuged at 2,500 g for 10 min at 4ºC to remove the platelets after being diluted with the same volume of PBS. The supernatant was then centrifuged at 16,500 g for 30 min at 4ºC to pellet EVs. The pellet was resuspended and washed with 0.2 μm-filtered PBS, stained with the FITC-conjugated F4/80 antibody (BioLegend, USA) at 1:50 for 30 min at 4ºC in dark, washed and examined by the ACEA NovoCyte flow cytometer (Agilent, USA). Collected mEVs in PBS were stained with fluorescence-conjugated antibodies for F4/80 and CD11b (both from BioLegend, USA) at 1:50 for 30 min at 4ºC in dark, washed and also examined by the ACEA NovoCyte flow cytometer (Agilent, USA). Collected macrophages after PA and mannose treatments were stained with a PE-conjugated antibody for CD86 (BioLegend, USA) at 1:50 for 30 min at 4ºC in dark, washed and examined by the ACEA NovoCyte flow cytometer (Agilent, USA).

High performance liquid chromatography (HPLC) analysis

D-mannose concentrations in serum and liver were examined by HPLC analysis. For serum, samples were added with distilled water at 1:2 (v/v), mixed by ultrasound treatment for 30 min on ice, and centrifuged at 12,000 g for 10 min at 4ºC. The supernatant was collected. For hepatic lysates, liver tissues were dissected at approximately 100 mg and homogenized in 500 μL distilled water on ice. The lysates were centrifuged at 12,000 g for 10 min at 4ºC, and the supernatant was collected. HPLC examination was performed on 10 μL samples at a

flow rate of 1.0 mL min^{-1} with HP-Amino columns (Sepax Technologies, USA).

Biodistribution analysis

Cy5.5-labeled mannose (Qiyue Biology, China) was oral gavaged at 10 mg, or DiR-labeled mEVs were intravenously injected at 200 μg on the basis of protein measurement, and mice were euthanatized after 24 h. The organs were harvested and imaged using the IVIS Lumina XRMS Series 2 instrument (PerkinElmer, USA) to assess the biodistribution of mannose, and the fluorescence intensity was quantified using the Living Image software (PerkinElmer, USA). The liver was then subjected to standard immunofluorescent (IF) staining for cellular uptake analysis, as stated below.

Histological analysis

At sacrifice, multiple organs were isolated and fixed overnight with 4% paraformaldehyde (PFA) (Saint-bio, China). Samples were dehydrated and embedded in paraffin, and 5 μm serial sections were prepared (RM2125; Leica, Germany). Sections then underwent hematoxylin and eosin (H&E) staining using a commercial kit (Beyotime, China). Hepatic steatosis was graded blindly based on the non-alcoholic fatty liver disease (NAFLD) activity score, which was performed by assessing the percentage of hepatocytes containing lipid droplets (S0: less than 5%; S1: 5-33%; S2: 34-66%; S3: greater than 66%).

Oil red O (ORO) and IF staining

At sacrifice, liver tissues were rapidly isolated, fixed overnight in 4% PFA, cryoprotected with 30% (w/v) sucrose (Solarbio, China), and embedded in optimal cutting temperature (OCT) compound (Sakura Finetek, USA). The specimens were snap-frozen and sectioned into 10 μm sagittal sections (CM1950; Leica, Germany). For ORO staining, liver sections were

immersed in 3 mg mL^{-1} ORO working solution (Aladdin, China) for 5 min and rinsed with distilled water. Sections were counterstained with the hematoxylin solution (Beyotime, China), mounted and photographed with a microscope (M205FA; Leica, Germany). Percentages of lipid droplet area were quantified using ImageJ 1.47 software (National Institute of Health, USA). For F-actin staining of hepatocyte borders, sections were probed with phalloidin conjugated to AlexaFluor-568 (Invitrogen, USA) according to the manufacturer's instructions, and counterstained with 4',6-diamidino-2-phenylindole (DAPI) (Abcam, UK). For IF staining of the protein markers, sections were treated with 0.3% Triton X-100 (Solarbio, China) diluted in PBS for 20 min at room temperature, blocked with 10% goat serum (Boster, China) for 1 h at room temperature, and stained with a rabbit anti-mouse inducible nitric oxide synthase (iNOS) primary antibody (Servicebio, China), a rat anti-mouse CD206 primary antibody (Bio-Rad, USA), or a rat anti-mouse F4/80 primary antibody (Abcam, UK) overnight at 4ºC at a concentration of 1:100. After washing with PBS, sections were then stained with a FITC-AffiniPure Goat Anti-Rabbit IgG secondary antibody (Yeasen, China) or a FITC-AffiniPure Goat Anti-Rat IgG secondary antibody (Yeasen, China) for 1 h at room temperature at a concentration of 1:200, and counterstained with DAPI (Abcam, UK). Fluorescent images were obtained using a confocal laser scanning microscope (Olympus, Japan).

Hepatic insulin signaling examination

Hepatic insulin signaling was evaluated by measuring insulin-stimulated protein kinase B (AKT) and adenosine 5'-monophosphate (AMP)-activated protein kinase alpha (AMPKα) phosphorylation. Briefly, mice were fasted for 8 h and then injected intraperitoneally with

recombinant human insulin (Novo Nordisk, Denmark) at 1 IU kg^{-1}. Mice were sacrificed at 15 min after injection, and liver tissues were collected and snap frozen with liquid nitrogen. The phosphorylation of AKT and AMPKα was determined by Western blot analysis, as stated below.

Micro-computed tomography (micro-CT) analysis

Femora were collected after sacrificing the mice and then fixed in 4% PFA overnight at 4°C. The fixed specimens were prepared as 1-cm length segments and scanned using a desktop micro-CT system (eXplore Locus SP, GE Healthcare, USA) with a resolution of 8 μm, voltage of 80 kV, and a current of 80 mA. Following three-dimensional image reconstruction, the regions of interest (ROIs) were selected. Specifically, trabecular bone ROIs were set from 0.3 to 0.8 mm away from the growth plates in the distal metaphysis, and cortical bone ROIs were defined in the midshaft, from 3.3 to 3.8 mm away from the growth plates. Data analysis was conducted using the VGStudio MAX software (Volume Graphics, Germany). The following trabecular and cortical bone parameters were measured: trabecular bone volume over tissue volume (Tb.BV/TV), trabecular bone mineral density (Tb.BMD), trabecular thickness (Tb.Th), trabecular number (Tb.N,), trabecular separation (Tb.Sp), and cortical thickness (Ct.Th).

Nanoparticle tracking analysis (NTA)

NTA was performed by a ZetaView instrument (Particle Metrix, Germany). Resuspended mEVs were diluted 50-fold in filtered PBS to achieve a final concentration of 3.2×10^{10} particle per mL. The capture length was 60 s with camera level set to 14 and detection threshold set to 3. The image of filtered PBS was taken to verify that the diluent had no particle in it. A total

of 1,498 frames were captured and analyzed. The ZetaView software (Particle Metrix, Germany) was used for capturing and data analysis.

Transmission electron microscopy (TEM) analysis

mEV pellet was resuspended in 2% PFA, and 20 μl mEVs were deposited on 200-mesh formvar-coated copper grids and dried at room temperature for 5 min. After removing excess suspension using filter paper, the mEVs were negatively stained with uranyl acetate (Sigma-Aldrich, USA) at room temperature for 2 min, washed with distilled water and dried. Imaging was performed under a FEI Tecnai G2 Spirit Biotwin TEM (Thermo Fisher Scientific, USA) operating at 100 kV, with a PHURONA camera (EMSIS, Germany) and RADIUS 2.0 software (EMSIS, Germany).

Metabolic assays of hepatocytes

For the glucose uptake assay, hepatocytes after overnight attachment were changed to DMEM medium with no D-glucose (Invitrogen, USA) for overnight incubation. A glucose uptake commercial kit based on the fluorescent glucose analog 2- (N-(7-nitrobenz-2-oxa-1,3-diazol-4-yl)amino)-2-deoxyglucose (2-NBDG) (Cayman Chemical, USA) was used to determine the glucose uptake rate. Glucose output of hepatocytes was also evaluated after overnight incubation in DMEM medium with no D-glucose (Invitrogen, USA). Hepatocytes were then treated with 2 mM sodium pyruvate (Sigma-Aldrich, USA) and 20 mM sodium lactate (Sigma-Aldrich, USA) for 4 h, and supernatant of the culture medium was collected and examined using a glucose determination commercial kit (Sigma-Aldrich, USA). For fatty acid uptake, a commercial kit (Abnova, USA) was used based on a fluorescent fatty acid substrate, which was added to cultured hepatocytes in BSA-free medium with the

fluorescence intensity being read immediately using the microplate reader (Synergy H1; Bio-Tek, USA) with the Gen5 software (Bio-Tek, USA) at 485/515 nm kinetically for 60 min, at an interval of 1 min. Lipid output of hepatocytes was also evaluated in BSA-free medium after adding 0.5 mM sodium acetate (Sigma-Aldrich, USA) for 2 h. Supernatant of the culture medium was collected and examined using the commercial kit for TG (Nanjing Jiancheng Biology Engineering Institute, China).

16S rRNA sequencing

Mouse fecal samples were collected with aseptic techniques, and total genomic DNA was extracted from the feces. PCR amplification of the V3-V4 region of bacterial 16S rRNA was performed using 341F and 805R primers. Quality control was performed on raw data to obtain high-quality clean data for subsequent analyses. Alpha diversity was calculated using the QIIME2 microbiome bioinformatics platform. Beta diversity was also calculated using QIIME2, and the graphics were plotted using R packages (v3.5.2). The sequence alignment was performed using Blast, and representative sequences were annotated using the SILVA database. All amplicon sequence variants (ASVs) identified in the 16S rRNA sequencing were listed in Table S1.

RNA sequencing analysis

Macrophages were treated with or without D-mannose at 25 mM for 48 h and then washed with PBS for 3 times. Total RNA was isolated using Trizol (Invitrogen, USA) according to the manual instruction. RNA sequencing libraries were generated with an insert size ranging from 100 to 500 bp, and sequenced using the BGISEQ-500 platform (Bioprofile, China). In the Linux environment, FastQC (version 0.12.1) was used for quality control filtering.

STAR (version 2.7.11a) was used to align clean reads to the reference genome. Gene abundance was represented as fragments per kilobase million (FPKM). In the R environment, DESeq2 package (version 1.4.5) was used for differentially expressed gene (DEG) (log2Fold change > 0.3 and adjusted p-value < 0.05) analysis. Pheatmap package (version 1.0.12) was used to generate the heatmap for the DEGs. The details of all the identified genes were listed in Table S2.

Network pharmacology

The target collection for D-mannose was obtained from online databases, including Swiss Target Prediction (PMID: 31106366), Similarity Ensemble Approach (PMID: 17287757), TargetNET (PMID: 27167132), BindingDatabase (PMID: 17145705), Therapeutic Target Database (PMID: 37713619), DrugBank (PMID: 18048412), STITCH (PMID: 18084021), ChEMBL (PMID: 21948594), and Pharmmapper (PMID: 20430828). Targets of type 2 diabetes (T2D) were identified using databases, such as MalaCards (PMID: 23584832), OMIM (PMID: 15608251), PharmGKB (PMID: 34387941), Therapeutic Target Database (PMID: 37713619), DigSeE (PMID: 23761452), DrugBank (PMID: 18048412), DisGenet (PMID: 31680165), and GeneCards (PMID: 20689021). The target genes of D-mannose in the interaction with T2D were determined by the intersection of D-mannose and T2D target gene sets. The Venn diagram was generated to visualize the overlapping genes using an online tool (https://bioinfogp.cnb.csic.es/tools/venny/index.html). Gene Oncology (GO) enrichment analysis was performed using the enrichment network tool. The top 20 GO enrichment items were listed according to the q-value (adjusted p-value), and the results were presented in a scatter plot using the Appyters network application.

RNA extraction and qRT-PCR analysis

Total RNA was isolated from freshly isolated liver tissues after homogenization under liquid nitrogen or from cultured RAW 264.7 using the commercial RNA isolation kits (Foergene, China) according to the manufacturer's instructions. The cDNA was synthesized using a commercial a PrimeScript™ RT Reagent Kit (Takara, Japan). Then, qRT-PCR was performed with a SYBR Premix Ex Taq II Kit (Takara, Japan) by a Real-Time System (CFX96; Bio-Rad, USA). Quantification was performed by using β-actin as the internal control and calculating the relative expression level of each gene with the $2^{-\Delta\Delta CT}$ method. Primers used in this study were listed in Table S3.

Western blot analysis

Whole lysates of liver tissues, RAW 264.7 cells or EVs were prepared using the RIPA Lysis Buffer (Beyotime, China). Proteins were extracted and the protein concentration was quantified using the BCA method (Beyotime, China). Equal amounts of protein samples were loaded onto SDS-PAGE gels and transferred to polyvinylidene fluoride (PVDF) membranes (Millipore, USA) which were blocked with 5% bovine serum albumin (BSA) (Gemini Bio, USA) in TBS for 2 h at room temperature. Then, the membranes were incubated overnight at 4°C with the following primary antibodies: anti-AMPKα (Cell Signaling Technology, USA; diluted at 1:1000), anti-p-AMPKα (Thr172) (Cell Signaling Technology, USA; diluted at 1:1000), anti-p-AKT (Ser473) (Cell Signaling Technology, USA; diluted at 1:1000), anti-AKT (Cell Signaling Technology, USA; diluted at 1:1000), anti-CD36 (Abmart, China; diluted at 1:1000), anti-CD9 (HuaBio, China; diluted at 1:1000), anti-CD63 (Thermo Fisher Scientific, USA; diluted at 1:1000), anti-CD81 (GeneTex, USA; diluted at 1:1000), anti-Cav-1 (Santa Cruz

Biotechnology, USA; diluted at 1:1000), anti-Mitofilin (Abcam, UK; diluted at 1:1000), anti-Golgin84/GOLGA5 (Novus Biologicals, USA; diluted at 1:1000), anti-GAPDH (Proteintech, China; diluted at 1:1000) and anti-β-actin antibodies (Proteintech, China; diluted at 1:2500). After washing with TBS containing 0.1% Tween-20, the membranes were incubated with horseradish peroxidase (HRP)-conjugated secondary antibodies (Signalway, China) for 1 h at room temperature. The protein bands were visualized using an enhanced chemiluminescence kit (Amersham Biosciences, USA) and detected by a gel imaging system (4600; Tanon, China). Grey values of bands were measured using the ImageJ software (NIH, USA).

Statistical analysis

Data were presented as mean ± standard deviation (SD) or as box (25th, 50th, and 75th percentiles) and whisker (range) plots of at least three independent experiments or three biological replicates. Data were analyzed by two-tailed unpaired Student's *t* test for two-group comparisons, one-way analysis of variation (ANOVA) followed by the Turkey's post-hoc test for multiple comparisons, or Kruskal-Wallis test for non-parametric comparisons using the Prism 8.01 software (GraphPad, USA). *P* values of less than 0.05 were considered statistically significant.

Note: *All antibody, Chemicals, Critical Commercial Assays, Medium, Cell Lines, Animals, Oligonucleotides, Recombinant DNA, Software and Algorithms information used in the paper can be found in Table S4.*

Supplementary Figures and Legends

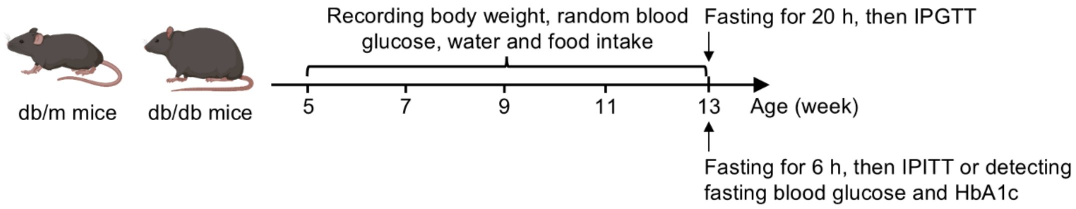

Figure S1. Schematic diagram showing the timeline of animal experiments (related to Figure 1). Mice were recorded for body weight, random blood glucose levels, water and food intake during the experimental period from 5-13 weeks of age. At 13-week old, mice were fasted for 20 h before intraperitoneal glucose tolerance test (IPGTT), or fasted for 6 h before intraperitoneal insulin tolerance test (IPITT) or detecting fasting blood glucose and glycated hemoglobin A1c (HbA1c).

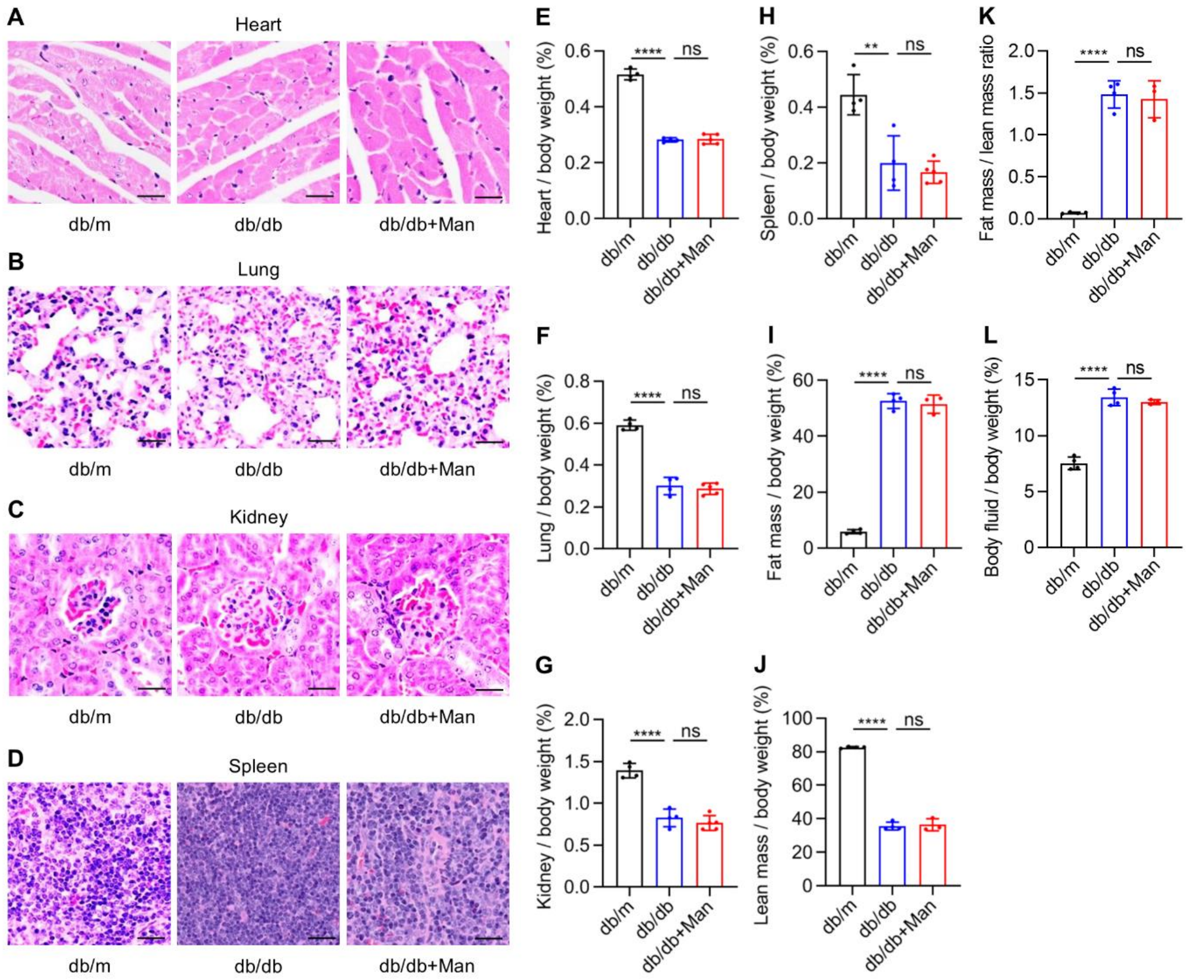

Figure S2. Multi-organ histological analysis reveals the safety of D-mannose therapy in db/db mice (related to Figure 2). (A) Hematoxylin and eosin (H&E) staining images showing the heart histology. Scale bars, 25 μm. (B) H&E staining images showing the lung histology. Scale bars, 25 μm. (C) H&E staining images showing the kidney histology. Scale bars, 25 μm. (D) H&E staining images showing the spleen histology. Scale bars, 25 μm. (E) Ratio of heart weight over body weight. n=4-5. (F) Ratio of lung weight over body weight. n=4-5. (G) Ratio of kidney weight over body weight. n=4-5. (H) Ratio of spleen weight over body weight. n=4-5. (I) Ratio of fat mass weight over body weight analyzed by magnetic resonance imaging (MRI). n=3-4. (J) Ratio of lean mass weight over body weight analyzed by MRI. n=3-4. (K) Ratio of fat mass weight over lean mass weight analyzed by MRI. n=3-4. (L) Ratio of body fluid weight

over body weight analyzed by MRI. n=3-4. Mean ± SD. **, $P < 0.01$; ****, $P < 0.0001$; ns, $P > 0.05$. One-way ANOVA with Turkey’s post-hoc test.

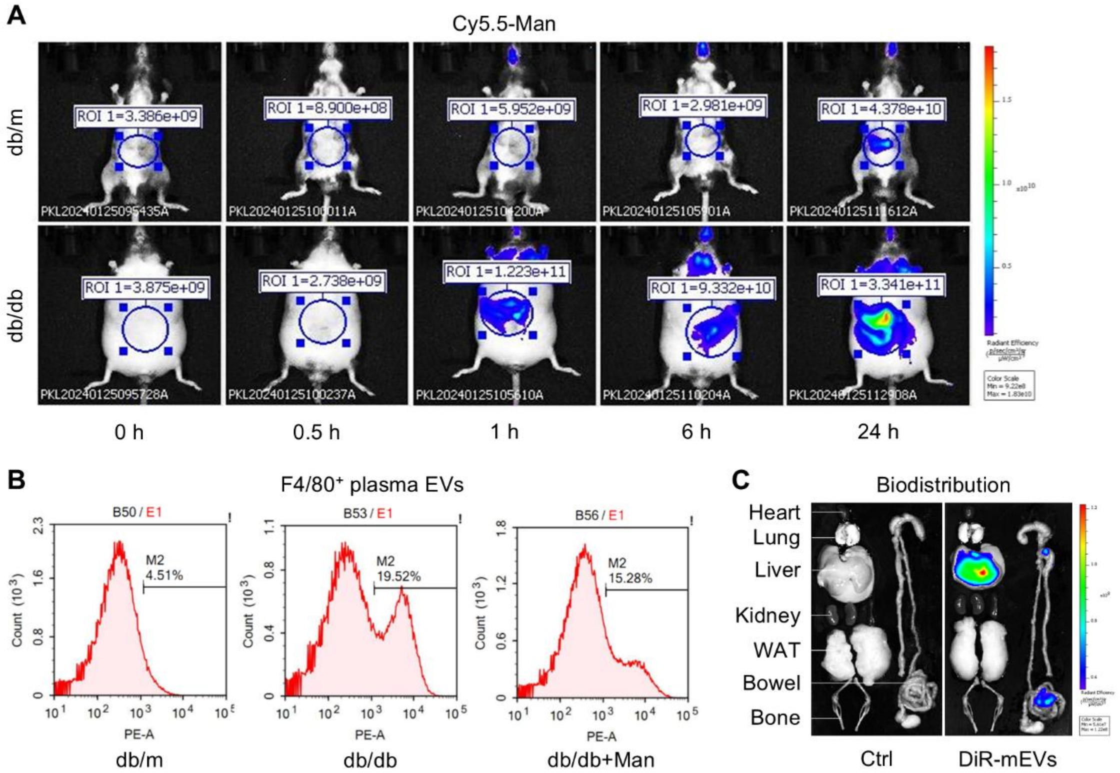

Figure S3. Biodistribution and flow cytometric analyses of exogenous mannose and macrophage-derived EVs (mEVs) (related to Figure 3 and Figure 5). (A) Biodistribution of Cy5.5-labeled fluorescent mannose (Man) after oral administration for different time points in db/m and db/db mice. (B) Flow cytometric analysis of F4/80^{+} plasma mEVs for quantification together with nanoparticle tracking analysis (NTA). (C) Biodistribution of DiR-labeled mEVs after intravenous administration for 24 h. WAT, white adipose tissue.

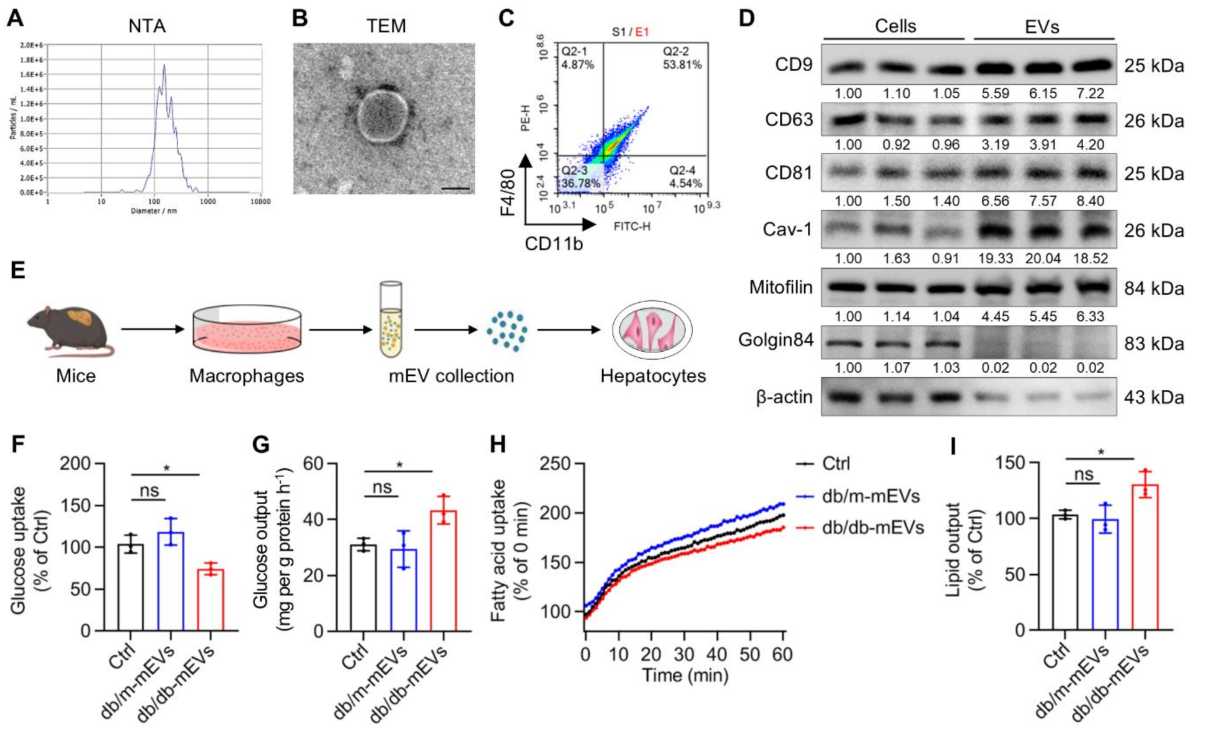

Figure S4. Macrophage-derived extracellular vesicles (mEVs) are characterized and regulate hepatocyte metabolism (related to Figure 5). (A) Nanoparticle tracking analysis (NTA) of mEVs showing the particle size distribution. (B) Transmission electron microscopy (TEM) analysis of mEVs. Scale bar, 50 nm. (C) Flow cytometric analysis of mEVs. (D) Western blot analysis of mEVs. Grey values of bands were relative to the far left ones and normalized to the respective β-actin band. (E) The diagram showing mEV collection and treatment of cultured hepatocytes. (F) Glucose uptake assay analyzed by 2-NBDG, a fluorescent deoxyglucose analog. n=3. (G) Glucose output from sodium pyruvate and sodium lactate. n=3. (H) Fatty acid uptake assay analyzed by fluorescent fatty acid substrate. n=3. (I) Lipid output from sodium acetate. n=3. Mean (H) or mean ± SD (F, G and I). *, $P < 0.05$; ns, $P > 0.05$. One-way ANOVA with Turkey's post-hoc test.

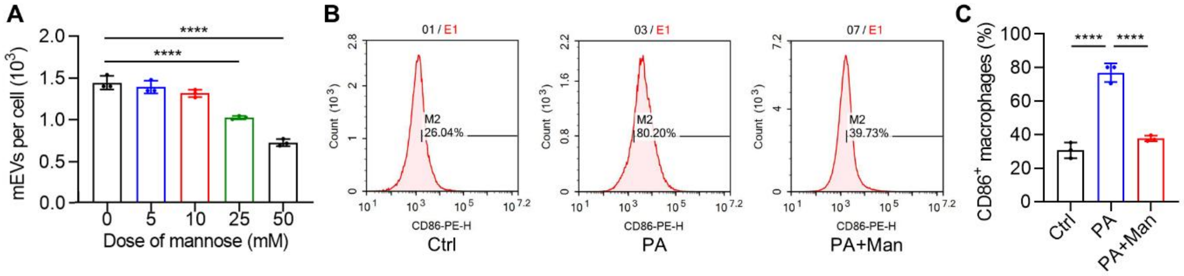

Figure S5. Effects of D-mannose on macrophage release of extracellular vesicles (EVs) and proinflammatory polarization (related to Figure 6). (A) Nanoparticle tracking analysis (NTA) quantification of macrophage-derived EVs (mEVs). Macrophages were treated with D-mannose for 48 h. n=3. (B) Flow cytometric analysis of CD86-positive percentages of macrophages after palmitic acid (PA) and mannose (Man) treatments. (C) Quantification of CD86-positive percentages of macrophages after *in vitro* treatment. n=3. Mean ± SD. ****, $P < 0.0001$. One-way ANOVA with Turkey's post-hoc test.

Supplementary Table Captions

Table S1. List of all amplicon sequence variants (ASVs) identified in the 16S rRNA sequencing.

Table S2. List of all genes identified in the transcriptomic analysis.

Table S3. List of primers used in this study.

Table S4. Key resources table.
