# Supplementary material for: D‐Mannose Alleviates Type 2 Diabetes and Rescues Multi‐Organ Deteriorations by Controlling Release of Pathological Extracellular Vesicles
Source: Exploration (Beijing). 2025 Aug 25;5(5):20240133. doi: 10.1002/EXP.20240133 (PMC12561340; doi:10.1002/EXP.20240133)
Supplement: Supplementary file 5 — exp270083‐sup‐0005 TableS4.docx [file EXP2-5-20240133-s002.docx]

**Table S4. Key resources table.**

| **REAGENT or RESOURCE** | **SOURCE** | **IDENTIFIER** |
| --- | --- | --- |
| Antibodies | | |
| APC anti-mouse CD4 | BioLegend | Cat# 100411, RRID: AB_312696 |
| PE anti-mouse CD8a | BioLegend | Cat# 100707, RRID: AB_312746 |
| FITC anti-mouse CD3 | BioLegend | Cat# 100203, RRID: AB_312660 |
| PE anti-mouse CD86 | BioLegend | Cat# 105008, RRID: AB_313151 |
| Alexa Fluor(R) 488 anti-mouse F4/80 | BioLegend | Cat# 123120, RRID: AB_893479 |
| Anti-CD9 Antibody [SA35-08] | HuaBio | Cat# ET1601-9, RRID: AB_3069620 |
| CD63 Polyclonal Antibody | Thermo Fisher Scientific | Cat# PA5-100713, RRID: AB_2850217 |
| CD81 antibody | GeneTex | Cat# GTX31381, RRID: AB_2887616 |
| Anti-iNOS Rabbit pAb | Servicebio | Cat# GB11119-100, RRID: AB_3064849 |
| Mouse Anti-Caveolin-1 Monoclonal antibody, Unconjugated, Clone 7c8 | Santa Cruz Biotechnology | Cat# sc-53564, RRID: AB_628859 |
| Mitofilin antibody [2E4AD5] | Abcam | Cat# ab110329, RRID: AB_10859613 |
| GOLGA5 Antibody | Novus Biologicals | Cat# NBP1-83352, RRID: AB_11002415 |
| Beta Actin antibody | Proteintech | Cat# 20536-1-AP, RRID: AB_10700003 |
| FITC anti-mouse/human CD11b | BioLegend | Cat# 101206 RRID: AB_312789 |
| PE anti-mouse F4/80 | BioLegend | Cat# 123110, RRID: AB_893486 |
| F4/80 antibody [CI:A3-1] | Abcam | Cat# ab6640, RRID: AB_1140040 |
| Phospho-Akt (Ser473) (D9E) XP® Rabbit mAb | Cell Signaling Technology | Cat# 4060 , RRID: AB_2315049 |
| Akt (pan) (C67E7) Rabbit mAb | Cell Signaling Technology | Cat# 4691, RRID: AB_915783 |
| CD36 antibody | Abmart | Cat# TU300524S |
| Anti-AMPK-alpha Antibody, Unconjugated | Cell Signaling Technology | Cat# 2532, RRID: AB_330331 |
| Rabbit Anti-AMPK-alpha, phospho (Thr172) Monoclonal Antibody, Unconjugated, Clone 40H9 | Cell Signaling Technology | Cat# 2535, RRID: AB_331250 |
| Rabbit Anti-TNFalpha Polyclonal, Unconjugated | Novus Biologicals | Cat# NBP1-19532, RRID: AB_164320 |
| Rat Anti-Mouse CD206 Monoclonal antibody, Unconjugated, Clone MR5D3 | Bio-Rad | Cat# MCA2235, RRID: AB_324622 |
| GAPDH antibody | Proteintech | Cat# 60004-1-Ig, RRID: AB_2107436 |
| FITC-AffiniPure Goat Anti-Rabbit IgG | Yeasen | Cat# 33107ES60 |
| FITC-AffiniPure Goat Anti-Rat IgG | Yeasen | Cat# 33307ES60 |
| Goat Anti-Mouse IgG Antibody, HRP conjugated Conjugated | Signalway | Cat# L3032, RRID: AB_895481 |
| Goat Anti-Rabbit IgG Antibody, HRP conjugated Conjugated | Signalway | Cat# L3012, RRID: AB_895483 |
| Chemicals, Peptides, and Recombinant Proteins | | |
| Red Blood Cell Lysis Buffer | Solarbio | Cat# R1010 |
| OptiLyse C, No-Wash Lysing Solution | Beckman Coulter | Cat# A11895 |
| Puromycin | Solarbio | Cat# P8230 |
| D-Mannose | Shanghai Yuanye Bio-Technology | Cat# S11071 |
| Palmitate acid | Kunchuang Biotechnology | Cat# SYSJ-KC002 |
| Murine M-CSF | PeproTech | Cat# 315-02-50UG |
| EGTA | Sigma-Aldrich | Cat# E3889 |
| Calcium chloride dihydrate | Sigma-Aldrich | Cat# 223506 |
| Liberase TM | Sigma-Aldrich | Cat# LIBTM-RO |
| Dexamethasone | Sigma-Aldrich | Cat# D4902 |
| Percoll | Sigma-Aldrich | Cat# P4937 |
| Sodium pyruvate | Sigma-Aldrich | Cat# P5280 |
| Sodium L-lactate | Sigma-Aldrich | Cat# L7022 |
| Sodium acetate | Sigma-Aldrich | Cat# S2889 |
| D-Mannose-6-phosphate, Disodium Salt hydrate | Aladdin | Cat# D350853 |
| a-D-Mannose-1-phosphate sodium salt | Aladdin | Cat# A334075 |
| D-Fructose-6-phosphate | Yingxinbio | Cat# 643-13-0 |
| MLS0315771 | MedChemExpress | Cat# HY-112945 |
| Tunicamycin | MedChemExpress | Cat# HY-A0098 |
| Heparin Solution | STEMCELL Technologies | Cat# 07980 |
| Recombinant Human Insulin | Novo Nordisk | Cat# ovolin^@^R Penfill |
| Glucose (AR) | Oubokai | N/A |
| Cy5.5-Mannose | Qiyue Biology | Cat# QY-C-CG1 |
| Sucrose | Solarbio | Cat# S8271 |
| Tissue-Tek® O.C.T. Compound | Sakura Finetek | Cat# 4583 |
| 4% General Purpose Tissue Fixative PFA | Saintbio | Cat# D16013 |
| Triton X-100 | Solarbio | Cat# T8200 |
| Mounting Medium with DAPI | Abcam | Cat# ab104139 |
| Oil red O | Aladdin | Cat# O104972 |
| Isopropyl Alcohol | Tianli | N/A |
| Methanol | Kemao | N/A |
| Ehanol | Fuyu | N/A |
| Uranyl acetate | Electron Microscopy Sciences | Cat# 22400 |
| Goat serum | Boster | Cat# AR0009 |
| Dimethyl sulfoxide | Solarbio | Cat# D8370 |
| Trizol | Invitrogen | Cat# 15596026 |
| Tris (Hydroxymethyl) Aminomethane | Solarbio | Cat# T8060 |
| Sodium dodecylsulfate | Solarbio | Cat# S8010 |
| Glycine | Solarbio | Cat# G8200 |
| Bovine serum albumin | Gemini Bio | Cat# 700-100P |
| 20 x TBS buffer | Coolaber | Cat# SL1327 |
| SWE Fast High Resolution Electrophoresis Buffer (Dry Powder) | Servicebio | Cat# G2081 |
| PVDF, 0.2 µm, 26.5 cm x 3.75 m | Millipore | Cat# ISEQ00010 |
| Critical Commercial Assays | | |
| Triglyceride assay kit | Nanjing Jiancheng Bioengineering Institute | Cat# A110-1-1 |
| Total cholesterol assay kit | Nanjing Jiancheng Bioengineering Institute | Cat# A111-1-1 |
| Nonesterified Free fatty acids assay kit | Nanjing Jiancheng Bioengineering Institute | Cat# A042-2-1 |
| AlexaFluor™ 568 phalloidin | Invitrogen | Cat# A12380 |
| PKH67 Red Fluorescent Cell Linker Kit | Sigma-Aldrich | Cat# PKH67GL |
| DiR Iodide (DiIC18(7)) | Yeasen | Cat# 40757ES25 |
| Mouse IL-10 ELISA Kit | Fankewei | Cat# F2176-B |
| Mouse TNF-α ELISA Kit | Fankewei | Cat# F2132-B |
| Glucose Uptake Cell-based Assay | Cayman Chemical | Cat# 600470 |
| Glucose (HK) Assay Kit | Sigma-Aldrich | Cat# GAHK20-1KT |
| Fatty Acid Uptake Assay Kit | Abnova | Cat# KA4084 |
| BCA Protein Quantitative Kit | Beyotime | Cat# P0010 |
| RIPA buffer (high) | Beyotime | Cat# P0013B |
| Animal Total RNA isolation Kit | Foergene | Cat# RE03014 |
| Cell Total RNA isolation Kit | Foergene | Cat# RE03113 |
| PrimeScript^TM^ RT reagent Kit with gDNA Eraser | Takara | Cat# RR047A |
| TB Green® Premix Ex Taq™ (Tli RNaseH Plus) | Takara | Cat# RR420A |
| Hematoxylin and Eosin Staining Kit | Beyotime | Cat# C0105M |
| Enhanced Chemiluminescence Kit | Amersham Biosciences | N/A |
| PAGE Gel Fast Preparation Kit | Epizyme Biotech | Cat# PG112 |
| Medium | | |
| DMEM, low glucose | Invitrogen | Cat# 11885-084 |
| DMEM, high glucose | Invitrogen | Cat# 11965-084 |
| DMEM, no glucose | Invitrogen | Cat# A1443001 |
| FBS, for macrophages | ExCell Bio | Cat# FND500 |
| FBS, for hepatocytes | Sigma-Aldrich | Cat# 17L624 |
| GlutaMAX™ Supplement | Invitrogen | Cat# 35050-061 |
| Penicillin-Streptomycin | Invitrogen | Cat# 15140-122 |
| PBS | Invitrogen | Cat# 10010023 |
| HBSS | Invitrogen | Cat# 14175-079 |
| William’s E Medium (WEM) | Invitrogen | Cat# 32551-087 |
| Experimental Models: Cell Lines | | |
| RAW264.7 | ATCC | Cat# TIB-71 |
| Experimental Models: Organisms/Strains | | |
| Mouse: BKS.Cg-*Dock7^m^* +/+ *Lepr^db^*/J mice | The Jackson Laboratory | Cat# JAX:000642, RRID: IMSR_JAX:000642 |
| Oligonucleotides | | |
| qRT-PCR primers | Invitrogen | N/A |
| Recombinant DNA | | |
| HBLV-m-CD36-3xflag-ZsGreen-PURO | Hanbio | Cat# LV79051616 |
| HBLV- ZsGreen-PURO | Hanbio | Cat# LV79051615 |
| Polybrene | Hanbio | Cat# 20230315 |
| Software and Algorithms | | |
| Gen5 | Bio-Tek | RRID: SCR_017317 |
| GraphPad Prism | GraphPad | RRID: SCR_002798 |
| ImageJ 1.47 | National Institute of Health | RRID: SCR_003070 |
| ZetaView Nanoparticle Tracking Analyzer | Particle Metrix | RRID: SCR_016647 |
| Bio-Rad CFX Maestro | Bio-Rad | N/A |
| Living Image | PerkinElmer | RRID: SCR_020397 |
| VGStudio MAX software | Volume Graphocs | RRID: SCR_017997 |
| Adobe Photoshop | Adobe Systems Software Ireland Ltd | N/A |
| Microsoft Office | Microsoft | N/A |
| OLYMPUS FLUOVIEW FV31S-SW | OLYMPUS | N/A |
| OlyVIA/XV Imaging | Olympus Soft Imaging Solutions GmbH | N/A |
| RADIUS 2.0 | EMSIS | N/A |
| Cytoscape (3.9.1) | https://cytoscape.org/ | RRID: SCR_003032 |
| QIIME2 | https://qiime2.org/ | RRID: SCR_021258 |
| BGISEQ-500 | https://www.bgi.com/global/company/careers/bgi-launches-its-desktop-sequencer-bgiseq-500/ | RRID: SCR_017979 |
| FastQC | https://github.com/s-andrews/FastQC | RRID: SCR_014583 |
| STAR | https://github.com/alexdobin/STAR | RRID: SCR_004463 |
| DESeq2 | https://bioconductor.org/packages/release/bioc/html/DESeq2.html | RRID: SCR_015687 |
| Pheatmap | https://cran.r-project.org/web/packages/pheatmap/index.html | RRID: SCR_016418 |
| R (4.3.0) | https://www.r-project.org/ | RRID: SCR_001905 |
